# Phenotypic Changes and Physiological Genetic Responses of *Oryza sativa* L. Roots Under Stress of Nanoplastics (NPs) and Cadmium (Cd) in Single and Combination Forms

**DOI:** 10.3390/genes17070835

**Published:** 2026-07-21

**Authors:** Haitao Liu, Hui Wang, Ling Liu, Ying Li, Chaoyu Lv, Yanhao Liu, Jingwen Gong, Lingling Liu

**Affiliations:** 1 School of Biological Engineering, Huainan Normal University, Huainan 232038, China; ht_liu@hnnu.edu.cn (H.L.); wh542968@sina.com (H.W.); ly3040468135@sina.com (Y.L.); l17201758168@sina.com (C.L.); 18110651252@163.com (Y.L.); gjw762835482@sina.com (J.G.); lll600lll600@sina.com (L.L.); 2 Key Laboratory of Bioresource and Environmental Biotechnology of Anhui Higher Education Institutes, Huainan Normal University, Huainan 232038, China

**Keywords:** nanoplastics, cadmium, rice seedlings, phytotoxicity, transcriptomic analyses, ecological risk

## Abstract

**Background/Objectives**: Both NPs and Cd alone exert adverse effects on plant growth by disrupting physiological processes and gene expression. However, the mechanisms underlying their combined effects on plant genetic responses remain incompletely understood. **Methods**: The rice seedlings were used as the experimental material, with the following six treatments established: CK (Control, no NPs and Cd), 10 and 100 mg·L^−1^ NPs, and 0.5 mg·L^−1^ Cd alone and combination. Seedlings at the three-leaf stage were treated in hydroponic culture for 7 days, after which root-development parameters, root viability, MDA and soluble sugar contents, and SOD and POD activities were measured, along with transcriptomic analysis. **Results**: The results show that root length, number of root tips, and root surface area were highest in CK compared with all NPs and Cd treatments, particularly. Significant differences were observed between the CK and both the high-concentration NPs and all Cd treatments groups (*p* < 0.05). Root growth progressively declined with increasing NP concentrations; the combination of 100 mg·L^−1^ NPs and 0.5 mg·L^−1^ Cd exhibited synergistic toxicity, which decreased root length, number of root tips, and root surface area by 14%, 22%, and 4% compared with 0.5 Cd, whereas 10 mg·L^−1^ NPs significantly alleviated Cd-induced root damage for the three root parameters above in the following order: 0.5 Cd < 0.5 Cd-10 NPs < 10 NPs. In terms of physiological changes, 10 mg·L^−1^ NPs reduced MDA levels and enhanced SOD and POD activities in roots exposed to Cd; in contrast, 100 mg·L^−1^ NPs exacerbated Cd-induced membrane peroxidation and decreased SOD and POD activities. In the high-concentration NPs and all Cd-treated groups, all aforementioned indicators exhibited significant differences compared with the CK (*p* < 0.05). Transcriptomic and WGCNA analyses revealed that the expression levels of *OsGRP162* (regulating resistance) and *OsCYP2* (inhibiting lateral root formation under overexpression) were significantly higher in 0.5 Cd-100 NPs compared with the other five treatments, while *OsTubA2* (positively regulating root length) exhibited a different pattern. Differentially expressed genes (DEGs) in experimental groups of 10 NPS_vs_0.5 Cd-10 NPS and 100 NPS_vs_0.5 Cd-100 NPS were predominantly enriched in glutathione metabolism and the MAPK signaling pathway, respectively. The key genes *OsMT4C*, *OsMT4B* and *OsYSL2* (associated with transmembrane signal transduction), and *OsABCB5* and *OsCUL1-3* (involved in negative regulation of root elongation) exhibited reduced expression levels in 0.5 Cd-10 NPs, whereas *OsYDA2* and *OsAGO1c* (related to antioxidant defense) showed upregulated expression. Conversely, the opposite gene expression patterns were observed in 0.5 Cd-100 NPs. **Conclusions**: These findings demonstrate that both NPs and Cd adversely affect rice seedlings; however, low concentrations of NPs mitigate Cd toxicity, while high concentrations exacerbate it. Therefore, to prevent elevated NP concentrations in plant growth environments, plastic usage and processing should be standardized.

## 1. Introduction

Since the 1950s, large-scale production has been achieved globally in industries such as manufacturing, leading to a rapid increase in plastic output [1]. However, plastic reuse rates remain low, with the majority of it being discarded into the environment. Under the effects of physical wear, chemical oxidation, biological contamination, heat, and sunlight exposure, larger plastics degrade into microplastics (MPs), with particle sizes ranging from 5 mm to 1 µm [2]. Studies indicate that the predominant types of microplastic polymers in the environment are polypropylene (PP), polyethylene (PE), polystyrene (PS), polyvinyl chloride (PVC), and polyamide (PA) [3,4]. After years of full environmental exposure, these MPs can further fragment into nanoplastic particles (NPs) that are smaller than 1 µm [5]. Due to their small size, NPs exhibit significantly enhanced surface reactivity, mobility, environmental bioavailability, and toxicity. Consequently, NPs have been recognized in recent years as a novel type of pollutant [6], with their presence detected in ecosystems such as oceans, rivers, and soils [7,8,9]. Research has extensively investigated the size distribution [10], properties [11], and abundance [12] of MPs. Through the analysis of 1039 papers from the Web of Science Core Collection and 135 sets of relevant data on soil, water, and atmosphere from typical regions in China, the results indicated that the soil contained a significant quantity of MPs, averaging 12,107.42 items/kg DW, while water contained averaging at 97,271.18 items/m^3^ [3]. Another study has shown that MPs were detected with a higher abundance in sediment (1356 ± 13 particles/kg) compared with water (582 ± 12 particles/m^3^), where sediments have the potential to act as long-term sinks for MPs [13]. According to statistics, extensive facility-based agricultural production has led to a significant increase in NPs in soils at 0–10 cm (8885 particles·Kg^−1^), with NPs content in soil ecosystems far exceeding those in the oceans [14]. Meanwhile, Recent studies have reported NP concentrations in aquatic environments ranging from 0.3 to 488 μg/L, while agricultural soils may contain up to 6.6 Mt of microplastics [15]. The impacts of NPs on soil properties and microbial growth have garnered widespread attention. Previous studies have demonstrated that NPs altered the structure and characteristics of farmland, impeded nutrient transport and, consequently, exerted negative effects on biodiversity [16,17,18]. Additionally, due to their small particle diameter, NPs are readily ingested by filter-feeding animals [19], annelids [20], and birds [21], subsequently migrating through the food chain and accumulating in higher organisms, leading to organ obstruction, mechanical damage, impaired immune function, or even mortality [22] and adversely affecting animal growth and reproduction [23]. Furthermore, NPs also exhibit detrimental effects on plants. Li et al. reported that NPs can form heterogeneous aggregates with microalgae and extracellular polysaccharides, suggesting that these aggregates serve as a critical pathway for NPs’ vertical transport, thereby inducing toxicity in microalgae and chlorella [24] and reducing photosynthesis [25]. NPs affect not only lower plants but also higher plants’ growth and physiological characteristics when they disrupt soil functions. Gao et al. demonstrated that NPs can be absorbed by plant roots into vascular tissues and, subsequently, reach aboveground organs [26], thereby reducing shoot-to-root biomass ratios and micronutrient contents [27] and damaging cell and organelle structures, ultimately inhibiting growth [28]. Thus, NPs pose a potential threat to producers in agricultural ecosystems.

In addition to NPs, heavy metals are also present in agricultural soils. Cadmium (Cd) is one of the most severely contaminating heavy metals in current soil environments, with excessive levels detected in certain crops that rely on soil as their growing medium [29]. Relevant research indicates that the global limit standard for Cd concentrations in soil is 0.35 mg/kg, and the threshold value for mean concentrations of Cd in natural background soil in China is 0.2 mg/kg [30]. The use of untreated industrial wastewater and sediment from sewage treatment plants for soil irrigation or fertilization can lead to significant concentrations of NPs in crop-growth environments [31,32], which makes the coexistence of Cd and NPs inevitable. NPs exhibit high hydrophobicity and a large specific surface area, enabling them to readily adsorb both organic and heavy metal pollutants. Extensive research has demonstrated the adsorption capacity of NPs for Cd [33,34] and synergistic or antagonistic effects of different concentrations NPs and Cd on plants [35]. Current studies primarily focus on the impacts of NPs, Cd alone, and combined stress conditions on crop growth, development, and physiological/biochemical processes [36,37]. As environmental pollution intensifies, the adverse effects of multiple combined stressors on crop growth and development have become increasingly pronounced, while transcriptomic analyses of crops under single-treatment conditions with NPs or Cd have been reported [38,39]. However, research investigating the genetic responses of crops under combined stress conditions remains limited. Will the invasion of nanoplastics affect the growth, development, and metabolic response of crops under Cd stress? Is the combined effect of nanoplastics and cadmium synergistic or antagonistic on crops, and does it have concentration-dependent tolerance? These issues are worth further research and exploration. Therefore, we hypothesize that the combined effects of NPs and Cd on rice roots are concentration-dependent: low-dose NPs mitigate Cd toxicity by activating antioxidant defenses (SOD/POD) and upregulating protective genes, whereas high-dose NPs exacerbate Cd-induced oxidative damage and membrane peroxidation by suppressing these pathways and disrupting root elongation-related gene expression, leading to synergistic toxicity. This study aims to investigate the genetic responses of rice seedlings to single and combined NPs and Cd stresses, as well as the mechanism by which different concentrations of nanoplastics alleviate or exacerbate Cd stress through analyzing root morphology and physiological changes in a plant rice model using transcriptomic approaches, thereby providing theoretical insights for environmental assessment.

## 2. Materials and Methods

### 2.1. Characteristic of the NPs

PS is an important model polymer for the investigation of the effects of microplastic (MP) and nanoplastic (NP) particles on living systems [40]. The red fluorescent microspheres (100 mg/10 mL) produced by PS-NPs (Tianjin BaseLine ChromTech Research Centre, Tianjin, China) were used in the present experiment. The characteristics of the PS-NPs were examined by TEM (Jeol 2100F, Jeol Co., Ltd., Akishima City, Japan) after diluting 100 times, and the average value of the diameters was 100 ± 2.25 nm (Figure S1) by measurement using Photoshop CS6 (PS-CS6).

### 2.2. Determination of Cd Concentration and Preparation of Stock Solution

Referring to the environmental quality standards for surface water (GB 3838-2002) and using a setup of 50 times the Cd limit of class V surface water [41], CdCl_2_·2.5 H_2_O (AR, Sinopharm Chemical Reagent Co., Ltd., Shanghai, China) was used in preparation of the Cd stocks.

### 2.3. Cultivation and Treatment of Rice Seedlings

*Oryza sativa* L. Jiayu 948 from the Zhejiang Academy of Agricultural Sciences was used as the research material. Plump and evenly sized kernels of Jiayu 948 were softened for 24 h with 0.1 mol L^−1^ HNO_3_ and soaked for 48 h with clear water at 35 °C in a thermostatic water bath. After germination, sprouted seeds were placed in a white porcelain dish and transferred to an artificial climate chamber (QHX-250BS-III, Shanghai Xinmiao Medical Machinery Manufacturing Co., Ltd., Shanghai, China) and cultured with 75% relative air humidity, a 28 °C/25 °C day/night regime, and a photoperiod of 15 h day/9 h night with active radiation of 350 μmol m^−2^s^−1^ until a 3-leaf stage appeared. Three hundred and sixty seedlings with the same height were selected, and 20 seedlings were transferred into each of the 18 Petri plates (diameter of 10 cm) containing 50 mL of the treatment solutions. The stock solutions of PS-NPs and/or Cd were diluted with Kimura B solution into the following treatment solutions: 0 mg·L^−1^ PS-NPs + 0 mg·L^−1^ Cd (CK, CK stand for control); 10 mg·L^−1^ PS-NPs (10 NP_S_); 100 mg·L^−1^ PS-NP_S_ (100 NP_S_); 0.5 mg·L^−1^ Cd (0.5 Cd); 0.5 mg·L^−1^ Cd + 10 mg·L^−1^ PS-NP_S_ (0.5 Cd-10 NPS); and 0.5 mg·L^−1^ Cd + 100 mg·L^−1^ PS-NP_S_ (0.5 Cd-100 NP_S_). Each treatment implemented in the culture room was conducted in triplicate, and the culture conditions were the same as described above, while the treatment solutions were removed and drained daily with syringes to disperse the NPS and replaced on the fourth day. The phenotype and physiological indexes of the *O. sativa* roots were determined after an exposure of 7 d.

### 2.4. Roots Development and Vitality Detection

Five seedlings were randomly selected from each Petri dish. After cleaning and unfolding, phenotypic pictures of the rice roots were acquired using a Color Flatbed Scanner (MRS-9600TFU2L, Shanghai Zhongjing Technology Co., Ltd., Shanghai, China) and then the length, diameter, and number of root tips and surface area and volume of each rice seedling root were calculated using a plant-root phenotyping analysis system (GXY-A, Zhejiang Top Cloud-Agri Technology Co., Ltd., Hangzhou, China). The triphenyltetrazolium chloride method (TTC) was used for assessing root-system vitality [42], and 1 cm of the root apex in red was excised, observed, and photographed using a stereomicroscope (Motic SMZ-140, Motic China Group Co., Ltd., Xiamen, China).

### 2.5. Determination of Malondialdehyde (MDA) Levels in Rice Seedlings Roots

The extraction and determination of malondialdehyde (MDA) in the roots were carried out according to the method of Li et al. [42]. The 0.5 g cut-and-shred root sample was ground with 10 mL trichloroacetic acid (TCA) and then centrifuged at 4000× *g* for 10 min. The mixture of 2 mL supernatant and 2 mL thiobarbituric acid (TBA) was boiled, rapidly cooled, and then centrifuged. Finally, the absorbances at 450 and 532 nm were immediately measured with a UV-visible spectrophotometer (LV-1600, Shanghai Mapada Instruments Co., Ltd., Shanghai, China). The calculation of the MDA content was based on the following formula: (6.45 × 10^−6^ × OD_532_ − 0.56 × 10^−6^ × OD_450_) × 0.5^−1^ × 1000^−1^.

### 2.6. Determination of Key Antioxidant Enzyme Activities in Rice Seedlings Roots

Crude protein in the roots was extracted with a buffer of Tris-HCl, and the nitrogen-blue tetrazole (NBT) photochemical reduction and guaiacol method were used in measuring activities for superoxide dismutase (SOD) and guaiacol peroxidase (POD) [43]. Detection of the isozymes of SOD and POD was conducted using the polyacrylamide gel electrophoresis (PAGE) method with the Mini-PROTEIN 3 electrophoresis system (Bio-Rad, Hercules, CA, USA), and the electrophoresis conditions were based on the protocol of Wang et al. [44] and Rong et al. [45] (voltage of 80 V for the separating gel, running buffer of 25 mmol/LTris, 192 mmol/Lglycin solution, pH 8.3).

### 2.7. Detection of Soluble Sugars

Referring to the experimental protocol described by Meng et al. [46], modified slightly, 0.1 g roots were crumbled, placed in a test tube containing 10 mL 80% ethanol, boiled for 30 min, and filtered in a 25 mL volumetric flask. Then, a mixture of 1 mL extract and 5 mL anthrone reagent was incubated for 10 min in a boiling water bath. The absorbance was measured at 625 nm, and the content was calculated according to a standard curve (Y = 0.0086X + 0.0186, R^2^ = 0.9995; X and Y represent the glucose concentration and OD_625_, respectively).

### 2.8. Transcriptome Analysis

A total of 0.5 g of roots from each sample was frozen with liquid nitrogen. The extraction (using TRIzol reagent), detection of RNA, and construction and quality inspection of the cDNA library were carried out sequentially. Agarose gel electrophoresis was used to analyze the integrity of the RNA and for the presence of DNA contamination; then the concentration and integrity of the RNA were measured and precisely detected using a Qubit^®^ 2.0 Fluorometer (Life Technologies brand, Carlsbad, CA, USA) and 2100 Bioanalyzer (Agilent Technologies, Inc., Santa Clara, CA, USA), respectively. The cDNA library was sequenced on an Illumina sequencing platform by Metware Biotechnology Co., Ltd. (www.metware.cn, Wuhan, China). The original data were filtered with fastp v 0.19.3 and the clean reads were obtained. After aligning the clean reads with the reference genome IRGSP-1.0_genome.fasta.gz (accessed on 20 July 2023, https://www.ncbi.nlm.nih.gov/sra/PRJNA988263) with HISAT2 [47], the gene expression quantification of the FPKM (fragments per kilobase of transcript per million fragments mapped) was calculated using feature Counts v1.6.2 [48]. Differentially expressed genes (DEGs) between the two groups were analyzed using DESeq2 under the conditions of a |log2 (fold change)| ≥ 1 and a false discovery rate (FDR) < 0.05 [49]. While the function annotation and enrichment analysis of the DEGs were from the NR (Non-Redundant Protein Sequence Database) and the KEGG (Kyoto Encyclopedia of Genes and Genomes), the screening of core module genes were conducted using weighted gene expression network analysis (WGCNA), and correlation networks and a heatmap of the hub genes in a certain color module were created with Cytoscape 3.10.4 and TBtool-II, respectively. The correlations between module genes and major growth physiological indicators were also calculated using TBtool (*p* < 0.05).

### 2.9. qRT-PCR Analysis

The extraction method used for the total RNA of the rice roots was the same as described above. After completing the purity and integrity determination, the RNA was transcribed into first-strand cDNA using the FastKing DNA kit (KR116) (Tiangen biotech Co., Ltd., Beijing, China). Quantitative reverse-transcription PCR (qRT-PCR) was performed using the Taq Pro Universal SYBR qPCR Master Mix (Vazyme Biotech, Nanjing, China) following the manufacturer’s protocol on a Step One Plus Real-Time PCR System (Applied Biosystems, Foster City, CA, USA). The relative expression levels of selected genes were calculated using the 2^−△△Ct^ method [50].

### 2.10. Statistical Analysis

Root-system phenotype parameters and physiological/biochemical index data were analyzed using one-way analysis of variance (ANOVA) in SPSS 24 with the results expressed as the mean ± standard deviation. Duncan’s test was employed to assess statistical differences (*p* < 0.05). Bar charts were generated using Origin 2024. Correlations among biological replicates, modular genes with different treatments, and physiological/biochemical indicators were determined using Pearson correlation.

## 3. Results and Discussion

### 3.1. Changes in Root Morphology and Vitality in Response to NP_S_ and Cd Single or Combined Stress

**Figure 1:**
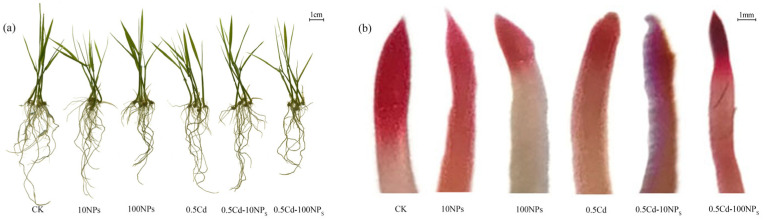
Effects of single or combined NPS and Cd stress on root morphology and vitality of *O. sativa* seedlings: (**a**) growth status of *O. sativa* seedlings root system; (**b**) changes in root tip vitality of *O. sativa* seedlings.

Roots play a key role in crop productivity as they sense change in external conditions and adapt to it by altering root structures [51]. In the present study, root growth exhibited consistent patterns: as the NP concentration increased, root development progressively declined after 7 days of treatment with NPs, Cd alone, and combined on rice seedlings (Figure 1a). Regarding root phenotypic parameters (Table 1), the root length, number of root tips, and surface areas of the three single-treatment groups were smaller than those in CK, and the corresponding developmental parameters of 100 NP_S_ and 0.5 Cd were significantly lower than those in 10 NP_S_ (*p* < 0.05). Under combined stress, the five measurement parameters of 0.5 Cd-10 NPs were higher than those in 0.5 Cd; however, the opposite situation occurred for 0.5 Cd-100 NP_S_, except for root diameter. The research findings indicate that low concentrations of PS-NPs (10 NP_S_) reduced Cd-induced damage to rice roots and promoted root development, whereas high concentrations of PS-NPs synergistically exacerbated the toxic effects on rice seedling. Previous experiments have confirmed that PS-NPs can penetrate the root systems of food crops, such as rice, fava beans, and wheat, with dose-dependent effects on root biomass and phenotypic traits [38,52,53]. In this study, the 100 NP_S_ treatment resulted in reductions of 44%, 32%, and 24% in root length, number of root tips, and root surface area, respectively, compared with the CK (Table 1), while no significant differences were observed among all phenotypic parameters under the 10 NP_S_ treatment. One possible reason is that low concentration PS-NPs directly enter rice roots via the explant pathway and aquaporins [38], without extensively covering the root surface to interfere with water and nutrient absorption. Cd is a toxic heavy metal that can enter rice through divalent ion transporters [39]. High concentrations of Cd in the plant-growth environment may impair root absorption of Fe^2+^, Zn^2+^, Mn^2+^, and Ca^2+^ [54], thereby inhibiting growth of both aboveground and underground tissues. Consequently, all phenotypic parameters of the root system in the single Cd treatment were lower than those in the CK (except root diameter). Furthermore, when high concentrations of Cd and PS-NPs are combined, the increased specific surface area of the PS-NPs enhances their adsorption and transport capacity for heavy metals, which are then delivered to rice roots via the exoplast pathway or ion channels. When root-cell-repair capacity exceeds the toxicity of accumulated heavy metals, root growth is severely compromised [55]. However, the coexistence of a low concentration of PS-NPs and Cd may lead to the competition for soil and root epidermal adsorption sites, leading to reduced unstable migration of Cd and decreased Cd ion uptake by roots [56], thereby mitigating Cd’s toxic effects. Thus, all root parameters under the 0.5 Cd-10 NP_S_ were superior to those of 0.5 Cd in this experiment.

As a vital vegetative organ, the root systems serve as a “bridge” for water and nutrient exchange between the aboveground and underground parts, directly influencing processes such as shoot growth and fruit development. Consequently, root system vitality is a core indicator for assessing plant physiological functions, and its decline directly reflects the detrimental effects of stress conditions on water absorption and nutrient transport [57,58]. TTC is a redox pigment that forms a colorless solution in water. It can be reduced by succinate dehydrogenase within root cells into a red, water-insoluble tritylamazine compound, which then deposits in root tissues. Therefore, it is commonly used to assess root vitality and reflect metabolic activity levels in roots. As shown in Figure 1b, the root systems vitality of five treatments whether with NP_S_ alone or in combination with Cd was lower than that of the CK. The tritylamazine compound content of 100 NP_S_ was less than 10 NP_S_, and dark red coloration and morphological abnormalities occurred at the root tips of 0.5 Cd-100 NP_S_. Possible reasons are the absence of water and mineral nutrients caused by NP_S_ coverage and Cd transfer, which impair photosynthesis, reduce organic matter transport from leaves to roots, and weaken root respiration [59]. In this study, root vitality measurements across all treatment groups showed significant correlations with the aforementioned morphological parameters. Root vitality was markedly reduced in the single cadmium treatment group compared with the control group, whereas it recovered somewhat in the composite treatment group involving low-concentration nanoplastics and cadmium, further confirming the mitigating effect of low-concentration PS-NPs on cadmium toxicity. Conversely, the high-concentration composite treatment group exhibited the lowest root vitality due to the synergistic toxicity of heavy metals and nanoparticles, indicating a clear dose-dependent relationship between pollutant concentration and the combined effects.

### 3.2. Effects of NPs and Cd Alone or Combined on Oxidative Stress

**Figure 2:**
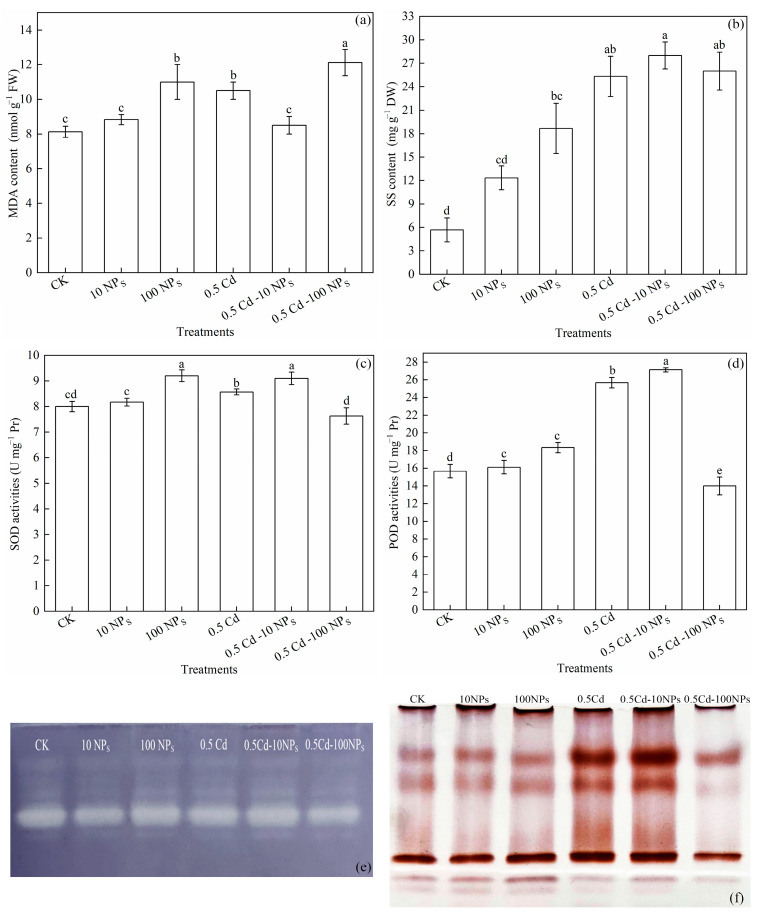
Changes in physiological and biochemical indicators of oxidative stress in *O. sativa* seedlings roots: (**a**) changes in MDA content; (**b**) changes in soluble sugar content; (**c**) changes in SOD activities; (**d**) changes in POD activities; (**e**) changes in CAT activities; (**e**) changes in SOD isoenzyme profile; (**f**) changes in POD isoenzyme profile. Different lowercase letters indicate significant differences among treatments (*p* < 0.05).

Usually, root system vitality is also affected by oxidative damage [60]. MDA is a membrane peroxidation product, and its level can be used to assess the extent of cell membrane damage [61]. In this experiment, MDA contents in the roots generally tended to increase with increasing concentrations of PS-NPs. The value of MDA under 0.5 Cd treatment was significantly higher than that in the CK and 10 NP_S_ treatments (*p* < 0.05). However, low concentrations of NP_S_ (10 mg L^−1^) reduced MDA levels under combined treatment with Cd, while it enhanced Cd-induced peroxidation in root cells at high concentrations (100 mg L^−1^), which increased by 49%, 10%, and 15% compared with CK, 100 NP_S_, and 0.5 Cd, respectively (Figure 2a). Therefore, high concentrations of PS-NPs increase toxicity when they are combined with Cd ions [62], and low concentrations of PS-NPs reduce the production of MDA caused by heavy metal stress [63]. The reason for this is that plants can produce soluble sugars (SS) through osmotic regulation and activation of antioxidant systems to regulate water potential, as well as generate antioxidant enzymes such as SOD and POD to scavenge reactive oxygen species (ROS) and reduce membrane peroxidation under certain adverse conditions [46]. Therefore, SS content and SOD and POD activity can reflect the resistance of the plant to stress. As shown in Figure 2b, the SS contents in the root systems of the five treatment groups were higher than that in the CK; rice exhibited superior osmotic regulation under Cd stress compared with PS-NPs. When in a composite state, both concentrations of NPs all enhanced the rice’s osmotic regulation capacity, but the difference in SS content with 0.5 Cd treatment was not significant (*p* > 0.05). Regarding the changes in the two isoenzymes, both NPs and Cd alone treatments resulted in higher SOD activity compared with the CK. However, significant differences were observed only between 100 NPs and 0.5 Cd versus CK (*p* < 0.05). When PS-NPs and Cd were applied jointly to rice seedlings, low concentrations (10 NPs) promoted SOD production and mitigated ROS-induced root damage caused by Cd stress, whereas high concentrations of PS-NPs (100 NPs) significantly reduced SOD levels and exacerbated Cd toxicity. The trend in changes in POD activity was largely consistent with those of SOD (except 0.5 Cd treatment) (Figure 2c,d). Isoenzyme profiles further confirmed the changes in SOD and POD activity (Figure 2e,f); the isozyme profiles revealed that a low concentration of NPs upregulated the band intensities of SOD and POD, enhancing superoxide and H_2_O_2_ scavenging to protect membranes. High concentrations of NPs combined with Cd, however, caused excessive ROS that inactivated these enzymes or suppressed their gene expression, reducing both SOD and POD activities. This decline compromised H_2_O_2_ detoxification and lignin synthesis, aggravating Cd uptake and lipid peroxidation. The differential isozyme responses underscore a threshold effect: mild stress activates protective isoforms, whereas severe stress overwhelms the antioxidant system, shifting from acclimation to toxicity. The present experimental results showed that *O. sativa* seedlings can tolerate stress caused by 100 mg L^−1^ PS-NPS, which is consistent with previous research findings [38]. When *O. sativa* seedlings were exposed to PS-NP_S_ combined with Cd, PS-NP_S_ under 10 mg L^−1^ relieved oxidative damage by increasing the activities of SOD and POD, but 100 mg L^−1^ PS-NP_S_ intensified the damage due to decreases in SOD and POD. That is, NP_S_ at higher concentrations can produce synergistic or additive effects with Cd, thus adversely affect plant growth and physiology [64]. The research results demonstrate that as the level of stress increases, the concentration of ROS also rises progressively; oxidative stress intensifies, and excessive ROS accumulation exceeds the clearance capacity of the defense enzyme system. The antioxidant enzyme system’s ROS scavenging mechanism may become disrupted, with antioxidant enzyme activity being severely inhibited or impaired.

### 3.3. Transcriptomic Analysis

Root architecture, as a critical morphological characteristic enabling plants to adapt to stressful environments, is genetically regulated [65]. The expression of defense-related genes in response to various stresses is induced by antioxidant enzymes and specific reactive oxygen species (ROS) acting as signaling molecules [66]. Consequently, this study conducted transcriptomic analysis to identify different expressed genes regulating root development and physiology under PS-NPs, Cd alone, or combined treatments.

#### 3.3.1. Transcriptome Sequencing and Data Analysis

Through high-throughput sequencing, the 18 samples in this study generated 44–60 million raw reads. These reads were filtered using fastp1.0, resulting in the retention of 96.5% clean reads, which contained 136.52 Gb of clean base pairs. Each sample achieved a minimum coverage of 6 Gb, with Q20 and Q30 base percentages exceeding 97% and 92%, respectively, and the GC content all surpassing 51% (Table S1). These values demonstrate that the sequencing data were of high quality. Additionally, the alignment efficiency, defined as the percentage of mapped reads relative to clean reads, directly reflects the utilization efficiency of transcriptomic data. An alignment efficiency exceeding 70% indicates successful alignment of sequencing reads to the reference genome [53]. The comparative efficiency of clean reads uniquely mapped to the rice reference genome in this experiment exceeded 87% (Table S2), with 93% of the mapped reads aligned to exon regions (Figure S2), indicating that the selected reference genome was appropriate. Additionally, the correlation coefficients for gene expression across all samples were greater than 0.95 (Figure S3), demonstrating strong inter-sample consistency and reliable assessment of DEGs.

#### 3.3.2. Transcript Expression and DEG Recognition

The number of expressed genes in the six different treatment groups is shown in Figure S4. The top ten genes with average expression levels in each treatment group were selected (Table S3). Among them, five genes, including *OsGRP162* (gene: *Os12t0632000-01*), *OsTubA2* (gene: *Os11t0247300-01*), *OsCYP2* (gene: *Os02t0121300-01*), *OsBBTI8* (gene: *Os01t0127600-01*), and putative (gene: *Os10t0465800-00*), coexisted. FPKM of *OsGRP162*, *OsCYP2,* and *OsBBTI8* were all the largest in 0.5 Cd-100 NPs. However, the expression pattern of *OsTubA2* was opposite. Since *OsBBTI8* and *Os10t0465800-00* are pseudogenes [67] and putative genes, respectively, they are not discussed in this context. To decipher the dose-dependent regulatory logic, we analyzed the top constitutively expressed genes. Notably, *OsGRP162* and *OsCYP2* exhibited a dramatic surge exclusively under 0.5 Cd-100 NPs. Given that *OsGRP162* is a stress-responsive RNA-binding protein likely induced by severe ROS/Ca^2+^ bursts [68], and *OsCYP2* is a known suppressor of lateral root initiation [69], their coordinated high expression directly accounts for the 22% reduction in root tip numbers (Table 1). Concurrently, *OsTubA2* (positively regulating root elongation) showed the lowest expression under this treatment. Thus, the synergistic repression of elongation-associated tubulins and overactivation of developmental suppressors collectively sculpt the inhibited root architecture under high-dose co-exposure.

GRP (glycine-rich RNA-binding protein) is an important structural protein of the plant cell wall, and the expression of its gene is tissue-specific, regulated by developmental stages and various environmental factors [70]. Previous studies have shown that *GRP2* can delay broccoli flower bud yellowing under cold shock treatment [71], and halophyte *Sporobolus virginicus* contained salt-tolerance genes *SvGRP1* and *SvGRP2* [72]. Genetic regulation studies in the plant rice model have demonstrated that *OsGRP3*/*OsGRP162* play a critical role in regulating heat tolerance during the seedling and reproductive stages [68]. Further, rice mutants with transforming Arabidopsis genes *AtGRP2* or *AtGRP7* exhibited increased yield under drought stress [73]. In our study, *OsGRP162* was specifically expressed in all six treatments. Under single stress, the FPKM value was highest under the 0.5 Cd treatment, and under combined stress, low concentrations of PS-NPs reduced *OsGRP162* expression, whereas high concentrations of PS-NPs significantly increased it. This heat-resistant gene exhibited upregulation under thermal stress simultaneously. In this experiment, the expression changes of *OsGRP162* under combined Cd and NPs treatments correlated with its heat stress response; the probable reasons is that Cd and NPs in high concentrations, like thermal stress, induce significant accumulation of intracellular reactive oxygen species (ROS), activate the Ca^2+^ or MAPK signaling pathways, and lead to activation of the *OsGRP162* promoter by the transcription factors WRKY and NAC, which are shared by different stress types [74]. *OsCYP2* is a proacyl protein gene in rice that plays a critical role in the initiation of lateral root development. Overexpression of *OsCYP2* leads to mutant rice plants exhibiting significantly shortened main roots and complete loss of lateral root formation capability [69]. *OsTubA2* (tubulin alpha-2 chain) can positively regulate root length [43]. Based upon the present findings, the high expression of *OsCYP2* and low expression of *OsTubA2* in 0.5 Cd-100 NPs are speculated to be major factors in reducing key root development parameters.

**Figure 3:**
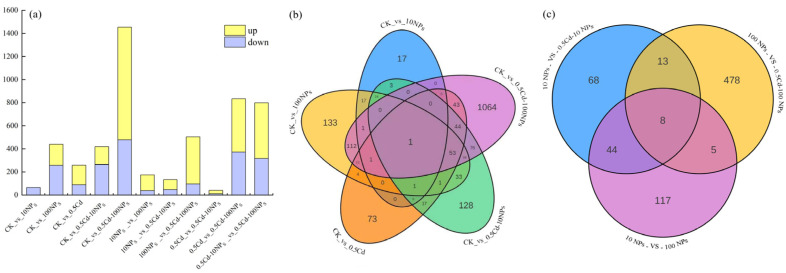
Changes in DEG expression: (**a**) upregulation and downregulation of DEGs; (**b**) coregulation of DEGs in comparison groups relative to the CK; (**c**) coregulation of DEGs in the comparison groups (among different NP treatments and NPs with Cd-NPs).

The distribution of DEGs across various treatment groups is shown in Figure 3a. The comparative results of the upregulated and downregulated DEGs between CK with NPs and Cd single/combined treatments, different concentrations of NP_S_ treatment, and NPs with the NP_S_ plus Cd composite treatment are presented in Table S4. The data indicate that the total number of DEGs and the counts of upregulated/downregulated genes for the eight comparison groups are 65 (0, 65), 440 (182,258), 259 (170,89), 419 (154,265), 1454 (975,479), 174 (135,39), 133 (85,48), and 504 (408,96). The CK_vs_0.5Cd-100NP_S_ group exhibited the highest number of DEGs; this result is further corroborated by the heat map (Figure S5). The top ten DEGs with the highest variation levels among the eight comparison groups are listed in Table S5. Subsequently, one DEG (gene: *Os02t0638650-01*: AP2 domain-containing protein) was identified in five of the comparison groups relative to the CK (Figure 3b) and eight DEGs in three of the comparison groups (Figure 3c). The functions, chromosomes, and sizes of these eight DEGs are provided in Table S6. The study results indicate that compared with the CK, no significant differences were observed in regulatory genes under either single NPs or Cd treatments or their combined effects. However, up to eight differential genes were identified across the comparison groups involving different NP concentrations and their combinations with Cd, which included *OsGRP1*, *NBS-LRR,* and holotricin-3 (disease-resistant genes) [75], except for four genes containing uncharacterized proteins and one novel 559. The dash indicates no known functional description.

Additionally, as shown in Table 2, the genes *OsMT4C*, *OsMT4B*, and *OsYSL2* were present in all comparison groups with CK and the three Cd-containing treatments. However, the log2FC value for CK_vs_0.5 Cd-10 NP_S_ was the lowest. These findings suggest that 10 mg·L^−1^ PS-NPs attenuates Cd transmembrane transport and decreases Cd toxicity in root cells, which is consistent with previous study findings [76]. But the gene (*OsYSL2*) transmembrane transporting Cd in the present experiment was different from *OsNRAMP5* [77] and *OsZIP16* [78] in rice roots, and the cause may be attributed to differences in rice varieties [79].

#### 3.3.3. KEGG Pathway Analysis

**Figure 4:**
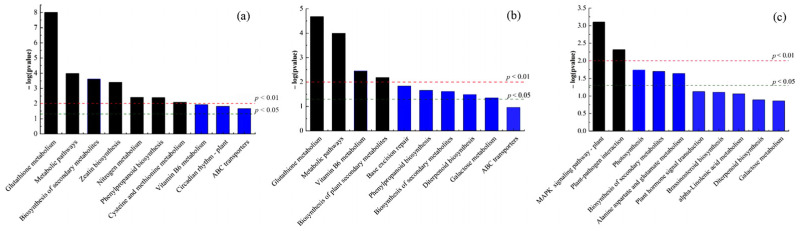
KEGG pathway enrichment analysis of DEGs identified in three comparison groups: (**a**) 10NPs_vs_100NPs; (**b**) 10NPs_vs_0.5Cd-10NPs; (**c**) 100NPs_vs_0.5Cd-100NPs.

By mapping differentially expressed genes to pathways in the KEGG database, the distribution of these genes across various pathways can be analyzed, thereby identifying pathways associated with specific biological processes [80]. Since the number of upregulated genes significantly exceeded that of downregulated genes among three of the comparison groups (Figure 3), a KEGG analysis was subsequently performed. Figure 4 shows that the top 10 pathways in the three comparison groups were significantly involved, five pathways (glutathione metabolism, metabolic pathways, phenylpropanoid biosynthesis, vitamin B6 metabolism, and ABC transporters) were enriched in 10 NP_S__vs_100 NP_S_ and 10 NP_S__vs_0.5 Cd-10 NP_S_. In addition to biosynthesis of secondary metabolites and diterpenoid biosynthesis, eight pathways (MAPK signaling pathway—plant, plant–pathogen interaction, photosynthesis, alanine aspartate and glutamate metabolism, plant hormone signal transduction, brassinosteroid biosynthesis, alpha-linolenic acid metabolism and galactose metabolism) were only in 100 NP_S__vs_0.5 Cd-100 NP_S_. In response to the stresses of NP_S_ single and combined treatment with Cd, *O. sativa* roots retained activity by regulating various physiological metabolisms; among them, nitrogen metabolism was only observed in the single NP treatment comparison group, while the root activity of the rice exposed to 0.5 Cd-100 NP_S_ mainly depended on an enhanced MAPK signaling pathway, plant–pathogen interaction, and photosynthesis to resist the toxicity of high concentrations of NP_S_ and Cd.

Both NPs and Cd are pollutants that affect plant growth. However, plants exhibit distinct genetic responses to these substances. Previous studies have investigated the independent effects of NPs and Cd on differential gene expression pathways. Zhou et al. reported that linoleic acid metabolism and phenylpropanoid biosynthesis were significantly enriched in rice roots under 10 mg·L^−1^ and 100 mg·L^−1^ PS-NPs stresses, respectively [38], while the enhanced activity in phenylpropanoid metabolism was found in rice root tips treated with 10 μM Cd [81]. In this experiment, compared with 10 NP_S_, the differentially expressed genes (DEGs) induced by 100 NP_S_ and 0.5 Cd-10 NP_S_ were predominantly enriched in glutathione metabolism but in the MAPK signaling pathway with 0.5 Cd-100 NP_S_ compared with 100 NP_S_. The difference arises from the distinct treatment conditions: this study focused on gene expression differences under different NP concentrations and between NP_S_ with NP_S_-Cd. However, previous studies compared the treatment group with the control group (CK).

The pathway shift from “Glutathione metabolism” (in 10 NPs_vs_0.5 Cd-10 NPs) to “MAPK signaling” (in 100 NPs_vs_0.5 Cd-100 NPs) reveals a fundamental defense transition. Under low-dose combined stress, enriched glutathione metabolism (e.g., *OsGSTU4*) efficiently scavenges lipid peroxides, preserving membrane integrity (lower MDA, Figure 2a). However, under high-dose co-exposure, overwhelming ROS levels surpass the glutathione pool’s capacity, damaging cell walls and releasing DAMPs, which activate MAPK and plant–pathogen interaction pathways. This triggers a hypersensitive response (HR)-like programmed cell death in root tips, sacrificing meristematic cells and drastically reducing root surface area. Therefore, the KEGG profiles illustrate a shift from adaptive resistance (low dose) to stress-induced senescence (high dose).

#### 3.3.4. Transcription Factors (TFs) and Weighted Gene Co-Expression Network Analysis (WGCNA)

**Figure 5:**
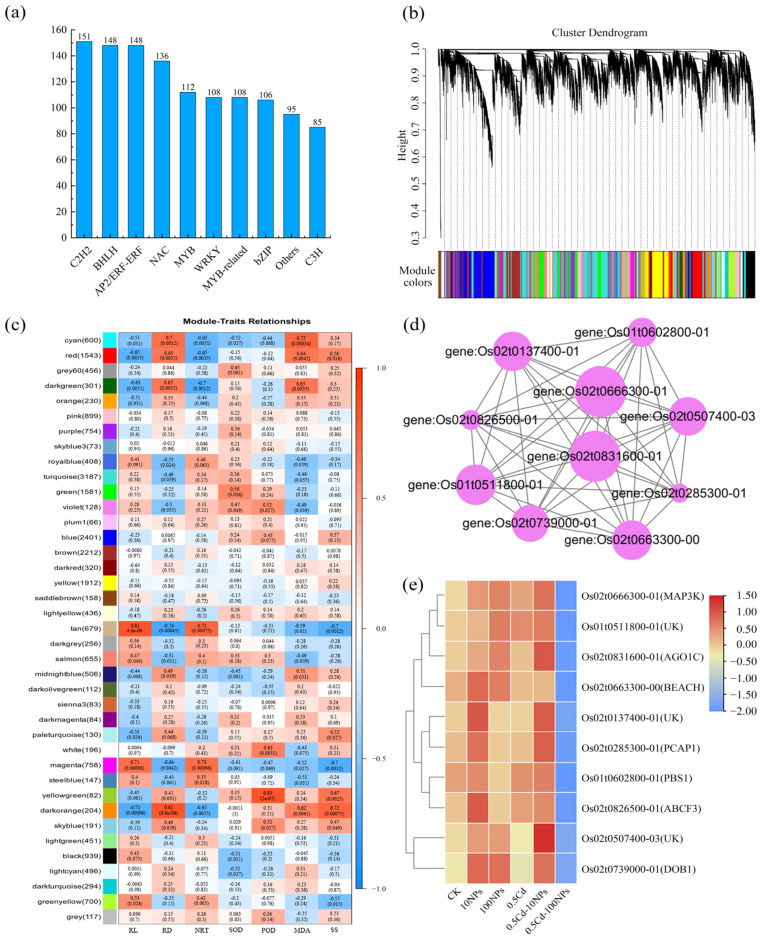
The results of the TF and WGCNA: (**a**) top 10 TFs; (**b**) hierarchical clustering tree showing co-expression modules identified by WGCNA; (**c**) correlation between module genes and growth physiological indicators (RL: root length, LD: root diameter, NRT: number of root tips); (**d**) correlation networks of hub genes in the violet module; (**e**) heatmap of hub genes in the violet module.

**Figure 6:**
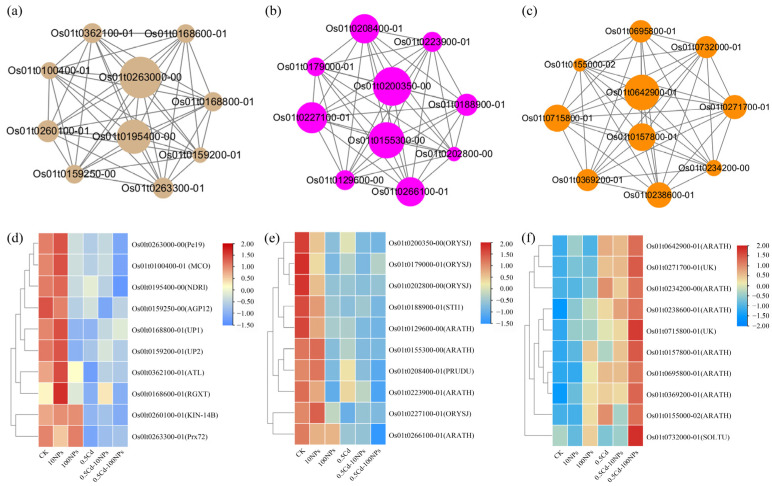
Hub genes in the different modules and their intra-modular relationships. Top 10 hub genes in the tan (**a**) and magenta (**b**) modules that are positively correlated with rice root growth. Top 10 hub genes in the dark-orange (**c**) module that are negatively correlated with rice root growth. (**d**–**f**) The corresponding heatmaps of the hub genes.

Transcription factors are a class of proteins that bind to DNA to regulate gene transcription, and changes in gene expression ultimately govern rice’s response to NPs or Cd [82]. As shown in Figure 5a, the top ten transcription factors (TFs) accounting for 43.07% of the total in this experiment are as follows: C2H2 (151), bHLH (148), AP2/ERF-ERF (148), NAC (136), MYB (112), WRKY (108), MYB-related (108), bZIP (106), Others (95), and C3H (85) families. Among them, all TFs, except Others, were involved in plant responses to stress conditions and growth development [46]. In WGCNA, thirty-nine highly correlated module genes with distinct color codes were identified (Figure 5b). The correlation co-efficient and significance between characteristic genes of the 39 modules and different parameters in growth and physiology are shown in Figure 5c. It is worth noting that violet module genes are significantly associated with SOD, POD activity, and MDA contents (*p* < 0.05), and the correlation coefficient and *p*-value are as follows: 0.47 (0.049), 0.52 (0.027), and-0.49 (0.039). Accordingly, tan module genes accompanied by magenta and dark-orange were significantly correlated with root development indicators (*p* < 0.01). Each module identified the top ten hub genes by standardization of K > 100 and edge weight > 0.2. Figure 5d,e shows that *OsYDA2* (gene: *Os02t0666300-01*) and *OsAGO1c* (gene: *Os02t0831600-01*) were key to regulating antioxidant enzyme activity, which were all upregulated except 0.5Cd-100NPs. But 10 hub genes in the dark-orange module played a contrary role in regulating the phenotype of the root system (Figure 6c,f). Among them, only *OsABCB5* (gene: *Os01t0695800-01*) and *OsCUL1-3* (gene: *Os01t0369200-01*) have been scientifically proven. WGCNA pinpointed hubs that mechanistically mediate the defense–growth trade-off. In the violet module (correlated with SOD/POD), *OsYDA2* (MAPKKK) and *OsAGO1c* were upregulated under 0.5Cd-10NPs, establishing a protective ROS-scavenging circuit. Conversely, in the dark-orange module (negatively correlated with root growth), *OsABCB5* and *OsCUL1-3* exhibit a strictly ascending expression order, as follows: 0.5 Cd-10 NPs < 0.5 Cd < 0.5 Cd-100 NPs. *OsABCB5* overexpression disrupts local auxin gradients essential for meristem activity [83], while *OsCUL1-3* accelerates ubiquitin-mediated degradation of cell-cycle regulators. We propose a hierarchical axis, as follows: high Cd-NPs stress → repression of *OsYDA2/AGO1c* (loss of defense) + activation of *OsABCB5/CUL1-3* (auxin collapse & cycle arrest) → synergistic root growth inhibition.

YDA is a gene belonging to the MAPKKK family. Drought stress activates the *OsCRK14* receptor kinase on rice root cell membranes, which phosphorylates the downstream cytoplasmic kinase *OsRLCK57*, thereby regulating the MAPK signaling pathway to enhance root resistance [84]. In contrast, *AGO* genes from the MIR168 family significantly enriched downstream differentially expressed genes associated with rice root resistance, such as ion transmembrane transport, plasma membrane components, and plant cell walls in the miR168-AGO1 downstream pathway [56]. *OsABCB5* negatively regulated the cold resistance of rice through the auxin signaling pathway [83], while overexpression of *OsCUL1-3* induced short roots in rice seedlings under salt stress [85]. These findings demonstrate that *OsABCB5* and *OsCUL1-3* suppressed root development under stress.

The experimental results demonstrate that under treatment with 0.5 Cd–100 NPs, both *OsYDA2* and *OsAGO1c* were downregulated within the MAPK-enriched pathway of root cells. However, they became upregulated under both 0.5 Cd and 0.5 Cd-10 NPs, with higher expression levels observed under the latter. The expression levels of *OsABCB5* and *OsCUL1–3* exhibited the following order: 0.5 Cd-100 NPs > 0.5 Cd > 0.5 Cd-10 NPs. These findings indicate that the combined exposure to high concentrations of Cd and NPs significantly enhances toxicity in rice seedlings, while 10 mg·L^−1^ of NPs primarily mitigates Cd toxicity through four specific genes.

#### 3.3.5. qRT-PCR Verification of Gene Expression

Eight DEGs, including one belonging to glycine-rich RNA-binding proteins, two related to protein folding and stress response, three belonging to redox and ROS scavenging, and two belonging to cell structure and cytoskeleton maintenance, were randomly selected to confirm the accuracy of the genes obtained by RNAseq. The designed primer sequences are shown in Table S7, and the actin gene served as the endogenous control for data normalization. Additionally, the relative expression levels of the eight genes are presented in Figure S6, which shows consistency with the trend in expression with Illumina sequencing. Beyond validation, qPCR captured the nuanced transcriptional dynamics of OsPrx5 (class III peroxidase). Cd treatment consistently repressed OsPrx5; however, this repression was slightly alleviated in 0.5 Cd-10 NPs (log2FC = −1.725) compared with 0.5 Cd-100 NPs (log2FC = −1.717). This subtle transcriptional relief, although modest, aligns precisely with the physiological reduction in MDA content (Figure 2a), confirming that low-dose NPs partially mitigate Cd-induced transcriptional suppression of apoplastic peroxidases, thereby reinforcing the antagonistic effect observed at the physiological level.

## 4. Conclusions

This study demonstrated that the combined toxicity of PS-NPs and Cd on rice roots exhibits a concentration-dependent effect. At 10 mg·L^−1^, NPs competitively occupy Cd-binding sites, leading to reduced expression levels of *OsMT4C*, *OsMT4B*, and *OsYSL2* genes in the cell membrane, thereby decreasing Cd transport and absorption and simultaneously upregulating *OsYDA2* and *OsAGO1c*, enhancing SOD and POD activities, and reducing MDA content, while downregulating *OsABCB5* and *OsCUL1-3* genes, and ultimately promoting root growth and mitigating Cd toxicity to some extent. However, for treatment with 100 mg·L^−1^ NPs combined with 0.5 mg·L^−1^ Cd, the expression trends of these genes were the opposite, with both phenotypic and growth physiological responses being weaker than those observed under the single Cd treatment. Thus, co-stress from high concentrations of NPs and Cd exacerbated the damage to rice roots. Therefore, it is imperative to reduce the use and discharge of non-degradable plastics and Cd and further strengthen environmental protection measures.

## Figures and Tables

**Table 1:** Root morphological parameters of *O. sativa* seedlings under PS-NPs alone and combined with Cd.

Treatments (mg·L^−1^)	Root Lenth (mm)	Root Diameter (mm)	Number of Root Tips	Surface Area (mm^2^)	Volume (mm^3^)
CK	256.0 ± 44.2 ^a^	0.49 ± 0.00 ^c^	22 ± 2.3 ^a^	396.6 ± 61.3 ^a^	79.1 ± 10.7 ^a^
10 NP_S_	213.7 ± 32.5 ^ab^	0.51 ± 0.05 ^c^	20 ± 3.2 ^ab^	342.4 ± 23.7 ^ab^	79.2 ± 6.6 ^a^
100 NP_S_	143.7 ± 11.9 ^c^	0.66 ± 0.04 ^ab^	15 ± 0.0 ^cd^	299.8 ± 22.5 ^b^	79.6 ± 11.1 ^a^
0.5 Cd	162.3 ± 18.6 ^c^	0.62 ± 0.03 ^b^	18 ± 1.0 ^bc^	341.3 ± 53.9 ^ab^	78.1 ± 22.8 ^a^
0.5 Cd-10 NP_S_	176.0 ± 10.6 ^bc^	0.67 ± 0.08 ^ab^	19 ± 0.6 ^bc^	342.2 ± 47.4 ^ab^	80.8 ± 21.2 ^a^
0.5 Cd-100 NP_S_	140.3 ± 6.1 ^c^	0.74 ± 0.08 ^a^	14 ± 1.0 ^d^	326.3 ± 32.5 ^ab^	76.9 ± 20.2 ^a^

Different letters following the mean ± SD in the same column indicate differences among the treatments. *n* = 3, *p* < 0.05.

**Table 2:** Expression of the relevant genes under Cd alone and Cd-NPs composite stress.

Gene	CK_vs_0.5 Cd		CK_vs_0.5 Cd-10 NPs		CK vs. -0.5 Cd-100 NPs		Related Functions
	log2FC	Express Trend	log2FC	Express Trend	log2FC	Express Trend	
*OsMT4C* (*novel.1479*)	4.19	up	3.496	up	4.039	up	Cd detoxification
*OsMT4B* (*Os12t0571000-01*)	3.64	up	3.169	up	3.977	up	Cd tolerance
*OsYSL2* (*Os02t0649900-01*)	2.01	up	1.847	up	4.016	up	Cd transport
*OsGSTU4* (*Os10t0528300-01*)	2.08	up	-	-	1.847	up	Antioxidation under Cd stress
*OsPrx5* (*0s05t0135500-01*)	-	-	−1.725	down	−1.717	down	Cd-induced ROS elimination

“-” Indicates that the gene was not detected in the alignment group.

## Data Availability

Data are contained within the article.

## Supplementary Materials

The following supporting information can be downloaded at: https://www.mdpi.com/article/10.3390/genes17070835/s1.
