# Supplementary material for: Phenotypic Changes and Physiological Genetic Responses of Oryza sativa L. Roots Under Stress of Nanoplastics (NPs) and Cadmium (Cd) in Single and Combination Forms
Source: Genes (Basel). 2026 Jul 21;17(7):835. doi: 10.3390/genes17070835 (PMC13409897; doi:10.3390/genes17070835)
Supplement: Supplementary file 1 [file genes-17-00835-s001.zip › Table S1.pdf]

Table S1 Sequencing data of rice root samples using in evaluation statistics

Sample	Raw Reads	Clean Reads	Clean Base(G)	Error Rate(%)	Q20(%)	Q30(%)	GC Content(%)
CK-1	54426758	52300880	7.85	0.03	97.62	93.42	52.96
CK-2	59491528	57918668	8.69	0.03	97.69	93.57	53.77
CK-3	57938130	55900914	8.39	0.03	97.47	93.09	53.38
10 NPs-1	47330452	45833362	6.88	0.03	97.54	93.24	53.5
10 NPs-2	56288890	54512242	8.18	0.03	97.6	93.27	51.64
10 NPs-3	54759142	53379100	8.01	0.03	97.82	93.8	51.79
100 NPs-1	59369272	57133070	8.57	0.03	97.57	93.24	51.64
100 NPs-2	44779284	42932708	6.44	0.03	98.03	94.39	52.05
100 NPs-3	52490584	50542082	7.58	0.03	97.71	93.65	53.38
0.5Cd-1	52705728	50990040	7.65	0.03	97.9	94.02	53.2
0.5Cd-2	52967800	51600734	7.74	0.03	97.95	94.11	54.01
0.5Cd-3	53663144	52085798	7.81	0.03	97.66	93.51	54.53
0.5Cd-10 NPs-1	47813304	46599982	6.99	0.03	98.02	94.18	51.86
0.5Cd-10 NPs-2	46231622	44912536	6.74	0.03	98.04	94.24	51.46
0.5Cd-10 NPs-3	49767354	47832864	7.17	0.03	97.98	94.14	51.86
0.5Cd-100 NPs-1	47033558	45050940	6.76	0.03	97.96	94.08	51.42
0.5Cd-100 NPs-2	45644406	43422316	6.51	0.03	97.31	92.66	52.96
0.5Cd-100 NPs-3	60123320	57040704	8.56	0.03	97.73	93.73	53.3
