# Supplementary material for: Phenotypic Changes and Physiological Genetic Responses of Oryza sativa L. Roots Under Stress of Nanoplastics (NPs) and Cadmium (Cd) in Single and Combination Forms
Source: Genes (Basel). 2026 Jul 21;17(7):835. doi: 10.3390/genes17070835 (PMC13409897; doi:10.3390/genes17070835)
Supplement: Supplementary file 1 [file genes-17-00835-s001.zip › Table S2.pdf]

Table S2 The proportion of sequencing reads successfully align to the reference genome

Sample	Total Reads	Reads mapped	Unique mapped	Multi mapped	Read1 mapped	Read2 mapped	'-' mapped
CK-1	52300880	48147981(92.06%)	46193948(88.32%)	1954033(3.74%)	23190874(44.34%)	23003074(43.98%)	23097141(44.16%)
CK-2	57918668	54262826(93.69%)	51980539(89.75%)	2282287(3.94%)	26076590(45.02%)	25903949(44.72%)	25988343(44.87%)
CK-3	55900914	52081697(93.17%)	49953269(89.36%)	2128428(3.81%)	25118143(44.93%)	24835126(44.43%)	24974022(44.68%)
10mPS-1	45833362	42605060(92.96%)	41080183(89.63%)	1524877(3.33%)	20642973(45.04%)	20437210(44.59%)	20540728(44.82%)
10mPS-2	54512242	50467456(92.58%)	48515752(89.00%)	1951704(3.58%)	24394249(44.75%)	24121503(44.25%)	24255329(44.50%)
10mPS-3	53379100	50054717(93.77%)	47739318(89.43%)	2315399(4.34%)	23955706(44.88%)	23783612(44.56%)	23865591(44.71%)
100mPS-1	57133070	53246883(93.20%)	51204661(89.62%)	2042222(3.57%)	25756885(45.08%)	25447776(44.54%)	25600744(44.81%)
100mPS-2	42932708	40113497(93.43%)	38540176(89.77%)	1573321(3.66%)	19375606(45.13%)	19164570(44.64%)	19272804(44.89%)
100mPS-3	50542082	46040764(91.09%)	44218527(87.49%)	1822237(3.61%)	22177853(43.88%)	22040674(43.61%)	22106782(43.74%)
0.5Cd-1	50990040	47358189(92.88%)	45571902(89.37%)	1786287(3.50%)	22822271(44.76%)	22749631(44.62%)	22784496(44.68%)
0.5Cd-2	51600734	48583125(94.15%)	46718257(90.54%)	1864868(3.61%)	23397817(45.34%)	23320440(45.19%)	23352676(45.26%)
0.5Cd-3	52085798	48312142(92.75%)	46215069(88.73%)	2097073(4.03%)	23195628(44.53%)	23019441(44.20%)	23100860(44.35%)
0.5Cd-10mPS-1	46599982	43490728(93.33%)	41815757(89.73%)	1674971(3.59%)	20945935(44.95%)	20869822(44.79%)	20905504(44.86%)
0.5Cd-10mPS-2	44912536	41495067(92.39%)	39832382(88.69%)	1662685(3.70%)	19955140(44.43%)	19877242(44.26%)	19911279(44.33%)
0.5Cd-10mPS-3	47832864	44821920(93.71%)	43080956(90.07%)	1740964(3.64%)	21580793(45.12%)	21500163(44.95%)	21537607(45.03%)
0.5Cd-100mPS-1	45050940	40939274(90.87%)	39404338(87.47%)	1534936(3.41%)	19745845(43.83%)	19658493(43.64%)	19697949(43.72%)
0.5Cd-100mPS-2	43422316	39266568(90.43%)	37844833(87.16%)	1421735(3.27%)	19029545(43.82%)	18815288(43.33%)	18929453(43.59%)
0.5Cd-100mPS-3	57040704	51723598(90.68%)	49790485(87.29%)	1933113(3.39%)	24964336(43.77%)	24826149(43.52%)	24891339(43.64%)
