# Supplementary material for: Phenotypic Changes and Physiological Genetic Responses of Oryza sativa L. Roots Under Stress of Nanoplastics (NPs) and Cadmium (Cd) in Single and Combination Forms
Source: Genes (Basel). 2026 Jul 21;17(7):835. doi: 10.3390/genes17070835 (PMC13409897; doi:10.3390/genes17070835)
Supplement: Supplementary file 1 [file genes-17-00835-s001.zip › Table S3--Table S4.pdf]

Table S3 The top ten genes of expression levels in different treatments

Treatments	Gene	FPKM	Description
CK	Os12t0632000-01	5844.24	glycine-rich RNA-binding protein 2
	Os01t0256500-02	5144.11	Os01g0256500, partial
	Os11t0247300-01	3294.98	tubulin alpha-2 chain
	Os05t0114700-02	3237.94	unknown protein
	Os02t0121300-01	2631.96	peptidyl-prolyl cis-trans isomerase
	Os01t0127600-01	2358.50	Bowman-Birk type bran trypsin inhibitor
	Os11t0703900-01	2252.29	heat shock cognate 70 kDa protein 2
	Os08t0116500-01	2163.46	60S acidic ribosomal protein P1
	Os12t0567700-01	2095.16	60S ribosomal protein L8
	Os10t0465800-00	2062.66	hypothetical protein EE612_051729, partial
10NPs	Os12t0632000-01	5624.50	glycine-rich RNA-binding protein 2
	Os11t0247300-01	3139.16	tubulin alpha-2 chain
	Os02t0121300-01	2981.48	peptidyl-prolyl cis-trans isomerase
	Os01t0256500-02	2703.52	Os01g0256500, partial
	Os05t0114700-02	2666.47	unknown protein
	Os10t0454200-01	2363.39	putative glycine-rich cell wall structural protein 1
	Os01t0127600-01	2021.21	Bowman-Birk type bran trypsin inhibitor
	Os10t0465800-00	1976.45	hypothetical protein EE612_051729, partial
	Os11t0703900-01	1969.45	heat shock cognate 70 kDa protein 2
	Os12t0567700-01	1960.53	60S acidic ribosomal protein P1
100NPs	Os12t0632000-01	5457.08	glycine-rich RNA-binding protein 2
	Os02t0121300-01	2989.30	peptidyl-prolyl cis-trans isomerase
	Os01t0127600-01	2640.16	Bowman-Birk type bran trypsin inhibitor
	Os05t0114700-02	2602.54	unknown protein
	Os11t0247300-01	2500.28	tubulin alpha-2 chain
	Os01t0256500-02	2474.08	Os01g0256500, partial
	Os11t0703900-01	2108.99	heat shock cognate 70 kDa protein 2
	Os10t0465800-00	2029.42	hypothetical protein EE612_051729, partial
	Os05t0160200-01	1933.75	ubiquitin-40S ribosomal protein S27a isoform X2
	Os12t0567700-01	1883.66	60S ribosomal protein L8
0.5Cd	Os12t0632000-01	5859.38	glycine-rich RNA-binding protein 2
	Os02t0121300-01	3425.26	peptidyl-prolyl cis-trans isomerase
	Os01t0256500-02	3113.89	Os01g0256500, partial
	Os11t0247300-01	3023.50	tubulin alpha-2 chain
	Os01t0127600-01	2508.72	Bowman-Birk type bran trypsin inhibitor
	Os05t0114700-02	2444.66	unknown protein
	Os08t0116500-01	2436.64	60S acidic ribosomal protein P1
	Os10t0465800-00	2397.54	hypothetical protein EE612_051729, partial
	Os05t0160200-01	2260.41	ubiquitin-40S ribosomal protein S27a isoform X2
	Os12t0567700-01	2228.57	60S ribosomal protein L8

0.5Cd-10NPs	Os12t0632000-01	5800.23	glycine-rich RNA-binding protein 2
	Os02t0121300-01	3705.61	peptidyl-prolyl cis-trans isomerase
	Os11t0247300-01	2734.65	tubulin alpha-2 chain
	Os10t0454200-01	2538.24	putative glycine-rich cell wall structural protein 1
	Os01t0256500-02	2182.28	Os01g0256500, partial
	Os01t0127600-01	2125.88	Bowman-Birk type bran trypsin inhibitor
	Os05t0114700-02	2111.40	unknown protein
	Os11t0703900-01	2070.74	heat shock cognate 70 kDa protein 2
	Os10t0465800-00	2048.34	hypothetical protein EE612_051729, partial
	Os10t0466700-01	1877.17	60S ribosomal protein L23
0.5Cd-100NPs	Os12t0632000-01	6328.50	glycine-rich RNA-binding protein 2
	Os02t0121300-01	4339.91	peptidyl-prolyl cis-trans isomerase
	Os01t0127600-01	3176.89	Bowman-Birk type bran trypsin inhibitor
	Os11t0247300-01	2512.65	tubulin alpha-2 chain
	Os05t0114700-02	2290.19	unknown protein
	Os08t0116500-01	2174.70	60S acidic ribosomal protein P1
	Os01t0256500-02	2074.86	Os01g0256500, partial
	Os10t0465800-00	2060.15	hypothetical protein EE612_051729, partial
	Os01t0328400-01	1989.55	Ubiquitin-40S ribosomal protein S27a-1, partial
	Os05t0160200-01	1917.50	ubiquitin-40S ribosomal protein S27a isoform X2

Table S4 Statistics of up-regulated and down-regulated DEGs

Group	down	up	total
CK_vs_10NPs	65	0	65
CK_vs_100NPs	258	182	440
CK_vs_0.5Cd	89	170	259
CK_vs_0.5Cd-10NPs	265	154	419
CK_vs_0.5Cd-100NPs	479	975	1454
10NPs_vs_100NPs	39	135	174
10NPs_vs_0.5Cd-10NPs	48	85	133
100NPs_vs_0.5Cd-100NPs	96	408	504
