# Supplementary material for: Phenotypic Changes and Physiological Genetic Responses of Oryza sativa L. Roots Under Stress of Nanoplastics (NPs) and Cadmium (Cd) in Single and Combination Forms
Source: Genes (Basel). 2026 Jul 21;17(7):835. doi: 10.3390/genes17070835 (PMC13409897; doi:10.3390/genes17070835)
Supplement: Supplementary file 1 [file genes-17-00835-s001.zip › Table S5.pdf]

Table S5. The top 10 differentially expressed genes with the highest degree of variation across the 8 comparison groups.

CK_vs_10MP$_S$					
ID	log2FoldChange	pvalue	padj	regulated	NR
Os01t0821300-01	-4.072495538	5.89455E-07	0.001650053	down	probable WRKY transcription factor 4 [Oryza sativa Japonica Group]
Os04t0301500-01	-3.694147703	4.57256E-07	0.001493322	down	hypothetical protein EE612_022967 [Oryza sativa]
Os01t0597600-01	-3.543942242	1.65541E-08	0.000107565	down	amino acid transporter AVT1I [Oryza sativa Japonica Group]
Os01t0656400-00	-3.498779348	2.17792E-05	0.014715948	down	transcription factor WRKY98 [Oryza sativa Indica Group]
Os07t0680600-01	-3.167225613	2.11528E-06	0.003768087	down	protein ENHANCED DISEASE RESISTANCE 4 [Oryza sativa Japonica Group]
Os08t0415600-00	-3.071453819	7.03391E-05	0.030628762	down	hypothetical protein EE612_044279 [Oryza sativa]
Os02t0764700-01	-2.882728304	1.26433E-05	0.011261159	down	ethylene-responsive transcription factor ERF109 [Oryza sativa Japonica Group]
Os08t0443800-01	-2.81591156	0.000177339	0.049015157	down	tetraspanin-8 [Oryza sativa Japonica Group]
Os02t0618400-02	-2.702051415	1.67492E-05	0.012623086	down	transcription factor MYB57 [Oryza sativa Japonica Group]
Os09t0325700-01	-2.660186779	1.22431E-08	0.000107565	down	probable protein phosphatase 2C 68 [Oryza sativa Japonica Group]
CK_vs_100MP$_S$					
ID	log2FoldChange	pvalue	padj	regulated	NR
Os08t0153750-00	7.580311018	4.2121E-07	0.000106469	up	Os08g0153800 [Oryza sativa Japonica Group]
novel.490	6.43185817	2.05447E-05	0.002109686	up	hypothetical protein DAI22_04g081000 [Oryza sativa Japonica Group]
Os03t0139500-01	6.406715372	0.000262628	0.016371192	up	uncharacterized protein LOC4331566 isoform X2 [Oryza sativa Japonica Group]
Os02t0765400-01	5.984677423	0.000846361	0.038823614	up	hypothetical protein EE612_013858, partial [Oryza sativa]
Os07t0638400-01	5.694278988	4.79974E-34	3.68012E-30	up	1-Cys peroxiredoxin B [Oryza sativa Japonica Group]
Os01t0718700-00	5.545536629	7.46276E-06	0.000940702	up	Os01g0718700, partial [Oryza sativa Japonica Group]
Os04t0339400-01	5.505177396	3.02933E-26	1.16134E-22	up	probable aldo-keto reductase 3 [Oryza sativa Japonica Group]
Os03t0226200-01	5.077210231	0.000662331	0.032655649	up	non-symbiotic hemoglobin 2 [Oryza sativa Japonica Group]
Os01t0869400-01	4.919305252	0.000354996	0.020465201	up	hypothetical protein EE612_007046, partial [Oryza sativa]
Os03t0233900-01	4.845418664	2.93412E-28	1.34981E-24	up	non-symbiotic hemoglobin 1 [Oryza sativa Japonica Group]
Os03t0369600-00	-3.931195763	0.000131833	0.009330518	down	hypothetical protein EE612_017626 [Oryza sativa]

Os04t0508400-00	-3.696757944	2.59805E-07	6.95722E-05	down	hypothetical protein EE612_024320 [Oryza sativa]
Os01t0821300-01	-3.630163834	3.57803E-06	0.000527576	down	probable WRKY transcription factor 4 [Oryza sativa Japonica Group]
Os10t0391400-01	-3.624083412	7.10033E-09	3.26644E-06	down	protein TIFY 11e [Oryza sativa Japonica Group]
Os08t0402001-00	-3.200368621	1.34407E-05	0.001493544	down	uncharacterized protein LOC107276925 [Oryza sativa Japonica Group]
Os04t0414500-01	-3.164850675	6.04299E-07	0.000141838	down	Os04g0414500 [Oryza sativa Japonica Group]
Os01t0656400-00	-3.135088567	8.72978E-06	0.001051321	down	transcription factor WRKY98 [Oryza sativa Indica Group]
Os01t0597600-01	-3.107159288	4.45552E-07	0.000111398	down	amino acid transporter AVT1I [Oryza sativa Japonica Group]
Os08t0415600-00	-2.991005195	9.47109E-05	0.007237678	down	hypothetical protein EE612_044279 [Oryza sativa]
Os06t0570566-00	-2.939875062	1.52276E-06	0.000283919	down	hypothetical protein EE612_034913 [Oryza sativa]
CK_vs_0.5Cd					
ID	log2FoldChange	pvalue	padj	regulated	NR
Os10t0361000-01	7.532558917	2.71587E-06	0.000644165	up	PLAT domain-containing protein 3 [Oryza sativa Japonica Group]
Os10t0150300-01	7.106987711	6.08005E-05	0.007903028	up	uncharacterized protein LOC4348119 [Oryza sativa Japonica Group]
Os07t0142100-01	6.986063727	4.80945E-05	0.006788408	up	hypothetical protein EE612_037058, partial [Oryza sativa]
Os03t0571800-00	6.583800743	0.000681772	0.043778345	up	hypothetical protein EE612_018536, partial [Oryza sativa]
Os04t0415401-01	6.264359897	6.72022E-05	0.008489152	up	hypothetical protein OsJ_14753 [Oryza sativa Japonica Group]
Os04t0107700-01	5.865108581	0.000185921	0.017675511	up	ervatamin-B [Oryza sativa Japonica Group]
Os01t0106400-01	5.73354691	1.87078E-23	1.07603E-19	up	isoflavone reductase homolog IRL [Oryza sativa Japonica Group]
Os06t0556150-00	4.81271798	9.98915E-05	0.011156324	up	putative amino acid carrier [Oryza sativa Japonica Group]
Os02t0571100-01	4.620088726	0.000169224	0.016638207	up	ent-copalyl diphosphate synthase 2 [Oryza sativa Japonica Group]
Os03t0663900-01	4.47662116	2.89618E-06	0.000673054	up	protein SCARECROW 2-like [Oryza sativa Japonica Group]
Os12t0637400-01	-4.272498291	3.91573E-08	1.80178E-05	down	expressed protein [Oryza sativa Japonica Group]
Os12t0575400-01	-4.037070412	3.43214E-08	1.6766E-05	down	uncharacterized protein LOC4352610 [Oryza sativa Japonica Group]
Os09t0409100-01	-3.439263749	8.02053E-08	3.35506E-05	down	MULTISPECIES: hypothetical protein [Bacilli]
Os06t0570566-00	-3.010586662	1.84636E-16	3.53992E-13	down	hypothetical protein EE612_034913 [Oryza sativa]
Os02t0184700-00	-2.990979222	6.76977E-05	0.008489152	down	cytochrome P450 71D7 [Oryza sativa Japonica Group]
Os08t0189300-01	-2.749469784	7.49627E-05	0.008936093	down	germin-like protein 8-4 precursor [Oryza sativa Japonica Group]
Os02t0649300-01	-2.667966804	0.000151226	0.015061757	down	homeobox-leucine zipper protein HOX24 [Oryza sativa Japonica Group]

Os12t0637200-01	-2.640164559	4.31248E-31	9.92171E-27	down	hypothetical protein EE612_061148, partial [Oryza sativa]
Os10t0507700-00	-2.528274913	1.78905E-05	0.003048942	down	hypothetical protein [Oryza sativa Japonica Group]
Os01t0375500-00	-2.517941275	3.38338E-07	0.000116181	down	hypothetical protein EE612_002721 [Oryza sativa]
CK_vs_0.5Cd-10MP$_S$					
ID	log2FoldChange	pvalue	padj	regulated	NR
Os10t0361000-01	7.926125176	1.61464E-06	0.000404383	up	PLAT domain-containing protein 3 [Oryza sativa Japonica Group]
Os10t0148100-01	7.754925823	1.65081E-05	0.002067218	up	proline-rich protein 2 [Oryza sativa Japonica Group]
Os07t0142100-01	6.983041548	7.26612E-08	3.23105E-05	up	hypothetical protein EE612_037058, partial [Oryza sativa]
novel.490	6.829087316	0.00028364	0.016658298	up	hypothetical protein DAI22_04g081000 [Oryza sativa Japonica Group]
Os06t0568600-01	5.520560403	9.28335E-05	0.007749994	up	ent-kaurene oxidase-like 5 [Oryza sativa Japonica Group]
Os02t0571100-01	5.268226215	1.12831E-05	0.001606842	up	ent-copalyl diphosphate synthase 2 [Oryza sativa Japonica Group]
Os10t0209700-01	4.71356085	0.000112229	0.008924624	up	heavy metal-associated isoprenylated plant protein 7 isoform X1 [Oryza sativa Japonica Group]
Os01t0106400-01	4.711833675	1.80667E-07	6.78716E-05	up	isoflavone reductase homolog IRL [Oryza sativa Japonica Group]
Os01t0342500-00	4.664084469	3.2796E-06	0.000700462	up	hypothetical protein EE612_002382, partial [Oryza sativa]
Os10t0113000-01	4.414784854	7.56608E-06	0.001221165	up	probable NAD(P)H-dependent oxidoreductase 1 [Oryza sativa Japonica Group]
Os12t0637400-01	-3.764936666	5.49651E-06	0.000950504	down	expressed protein [Oryza sativa Japonica Group]
Os06t0570566-00	-3.456379887	6.0351E-10	4.69639E-07	down	hypothetical protein EE612_034913 [Oryza sativa]
Os04t0659300-01	-3.150587109	4.37069E-06	0.000823567	down	cysteine-rich receptor-like protein kinase 6 [Oryza sativa Japonica Group]
Os02t0649300-01	-3.005878421	1.89597E-05	0.002290526	down	homeobox-leucine zipper protein HOX24 [Oryza sativa Japonica Group]
Os02t0184700-00	-2.889655364	8.71397E-05	0.007359252	down	cytochrome P450 71D7 [Oryza sativa Japonica Group]
Os12t0637200-01	-2.766596605	1.10141E-24	1.19993E-20	down	hypothetical protein EE612_061148, partial [Oryza sativa]
Os08t0415600-00	-2.763123907	0.001523903	0.049484822	down	hypothetical protein EE612_044279 [Oryza sativa]
Os02t0663900-01	-2.730634516	3.65115E-06	0.000736619	down	FT-interacting protein 1 [Oryza sativa Japonica Group]
Os01t0656400-00	-2.728340043	9.77054E-05	0.00809469	down	transcription factor WRKY98 [Oryza sativa Indica Group]
Os01t0895200-01	-2.720196004	1.15938E-07	4.51104E-05	down	hypothetical protein EE612_007351 [Oryza sativa]
CK_vs_0.5Cd-100MP$_S$					
ID	log2FoldChange	pvalue	padj	regulated	NR

Os10t0361000-01	9.134283923	7.94579E-14	1.09389E-11	up	PLAT domain-containing protein 3 [Oryza sativa Japonica Group]
novel.490	8.699156023	6.65643E-13	7.96437E-11	up	hypothetical protein DAI22_04g081000 [Oryza sativa Japonica Group]
Os10t0451700-01	8.111142995	1.33997E-08	8.03081E-07	up	glycine-rich cell wall structural protein [Oryza sativa Japonica Group]
Os03t0571800-00	7.457825021	1.9707E-09	1.38148E-07	up	hypothetical protein EE612_018536, partial [Oryza sativa]
Os05t0270500-01	7.184791733	9.75615E-08	4.99084E-06	up	probable ribonuclease P/MRP protein subunit POP5 [Oryza sativa Japonica Group]
Os08t0153750-00	7.179463194	9.01763E-08	4.67301E-06	up	Os08g0153800 [Oryza sativa Japonica Group]
Os01t0738400-01	6.817416254	5.97395E-07	2.48338E-05	up	zinc finger CCCH domain-containing protein 10 [Oryza sativa Japonica Group]
Os03t0226200-01	6.610009951	2.2974E-06	8.28116E-05	up	non-symbiotic hemoglobin 2 [Oryza sativa Japonica Group]
Os04t0125700-01	6.563193224	3.19157E-06	0.000110625	up	L-type lectin-domain containing receptor kinase IX.1 [Oryza sativa Japonica Group]
Os09t0241700-01	6.306709572	1.94317E-05	0.000512553	up	uncharacterized protein LOC112270781 [Brachypodium distachyon]
Os12t0555100-01	-6.523198108	1.55822E-06	5.8473E-05	down	major pollen allergen Cor a 1 isoforms 5, 6, 11 and 16 [Oryza sativa Japonica Group]
Os10t0550800-00	-5.418609712	4.21342E-08	2.33712E-06	down	hypothetical protein EE612_052659, partial [Oryza sativa]
Os01t0773350-01	-4.695426275	0.000143394	0.002625787	down	hypothetical protein OsJ_03631 [Oryza sativa Japonica Group]
Os01t0550800-01	-4.541931474	3.59863E-08	2.0306E-06	down	uncharacterized protein LOC4325841 [Oryza sativa Japonica Group]
Os06t0570566-00	-4.341142216	8.19112E-36	7.48766E-33	down	hypothetical protein EE612_034913 [Oryza sativa]
Os03t0369600-00	-4.226273572	6.39313E-05	0.001362459	down	hypothetical protein EE612_017626 [Oryza sativa]
Os12t0104766-00	-4.189106789	0.001473159	0.016807836	down	hypothetical protein EE612_057292 [Oryza sativa]
Os11t0134100-00	-4.059438909	0.000175896	0.003099261	down	Os11g0134100 [Oryza sativa Japonica Group]
Os06t0570600-00	-3.851308603	2.03586E-09	1.4228E-07	down	PUTATIVE PSEUDOGENE: RecName: Full=Ent-kaurene oxidase-like protein 1; Short=OsKOL1; AltName: Full=Cytochrome P450 701A7 [Oryza sativa Japonica Group]
Os09t0548700-01	-3.833608274	4.65864E-05	0.001035642	down	probable flavin-containing monooxygenase 1 [Oryza sativa Japonica Group]
$10MP_S$_vs_$100MP_S$					
ID	log2FoldChange	pvalue	padj	regulated	NR
Os04t0339400-01	7.947526044	2.80517E-15	2.80724E-12	up	probable aldo-keto reductase 3 [Oryza sativa Japonica Group]
Os08t0153750-00	7.495184085	6.85461E-07	0.000228656	up	Os08g0153800 [Oryza sativa Japonica Group]
Os07t0638400-01	6.534384388	1.31208E-52	7.55006E-49	up	1-Cys peroxiredoxin B [Oryza sativa Japonica Group]
Os11t0226800-00	6.25444766	0.000139315	0.01920133	up	NBS-LRR-like protein [Oryza sativa Japonica Group]
Os02t0770800-01	6.15502305	9.88717E-70	1.13786E-65	up	nitrate reductase [NAD(P)H]

Os09t0367700-01	5.784809954	2.96701E-73	6.82916E-69	up	hypothetical protein EE612_047349, partial [Oryza sativa]
Os01t0718700-00	5.450771915	1.33786E-05	0.002926311	up	Os01g0718700, partial [Oryza sativa Japonica Group]
Os02t0112100-01	5.346466835	5.88583E-05	0.009961341	up	high-affinity nitrate transporter 2.1 [Oryza sativa Japonica Group]
Os07t0638300-01	4.842729973	1.49681E-09	9.06636E-07	up	1-Cys peroxiredoxin A [Oryza sativa Japonica Group]
Os10t0528300-01	4.767285458	1.43094E-64	1.09787E-60	up	putative glutathione S-transferase OsGSTU4 [Oryza sativa Japonica Group]
Os08t0518900-01	-4.043509696	5.09468E-05	0.008883663	down	xylanase inhibitor protein 1-like [Oryza sativa Japonica Group]
Os06t0570566-00	-3.270379861	0.000349298	0.03756917	down	hypothetical protein EE612_034913 [Oryza sativa]
Os01t0193250-00	-3.190543025	0.000124979	0.017540483	down	hypothetical protein OsI_00742 [Oryza sativa Indica Group]
Os04t0481700-01	-3.15804272	1.68736E-05	0.003530714	down	MDR-like ABC transporter [Oryza sativa Japonica Group]
Os06t0570600-00	-2.908169019	0.000460804	0.046930621	down	PUTATIVE PSEUDOGENE: RecName: Full=Ent-kaurene oxidase-like protein 1; Short=OsKOL1; AltName: Full=Cytochrome P450 701A7 [Oryza sativa Japonica Group]
Os12t0637200-01	-2.284624869	2.61445E-07	9.86504E-05	down	hypothetical protein EE612_061148, partial [Oryza sativa]
Os04t0609300-01	-2.225245604	0.000183422	0.023324949	down	hydroxycinnamoyltransferase 4-like [Oryza sativa Japonica Group]
Os08t0189300-01	-2.116731637	2.61982E-05	0.005198314	down	germin-like protein 8-4 precursor [Oryza sativa Japonica Group]
Os11t0501500-01	-2.001418715	1.95027E-05	0.003937666	down	Os11g0501500, partial [Oryza sativa Japonica Group]
Os11t0643400-00	-1.864139397	3.90552E-05	0.007203151	down	hypothetical protein EE612_056864 [Oryza sativa]
10mMP$_S$_vs_0.5Cd-10MP$_S$					
ID	log2FoldChange	pvalue	padj	regulated	NR
Os07t0142100-01	9.112194474	9.93493E-05	0.018262145	up	hypothetical protein EE612_037058, partial [Oryza sativa]
Os11t0206000-01	7.474803841	1.46999E-05	0.003868912	up	hypothetical protein OsJ_14266 [Oryza sativa Japonica Group]
novel.1366	6.308926059	5.64111E-05	0.012097569	up	Os11g0691290 [Oryza sativa Japonica Group]
Os11t0226800-00	5.681966867	0.000372357	0.045439397	up	NBS-LRR-like protein [Oryza sativa Japonica Group]
Os01t0800900-01	4.715397866	2.28387E-27	5.28967E-23	up	hypothetical protein EE612_006309, partial [Oryza sativa]
novel.559	4.5439931	5.87771E-20	4.53778E-16	up	--
Os05t0404901-00	4.528811474	0.000107055	0.019073085	up	hypothetical protein [Oryza sativa Japonica Group]
novel.758	4.320987739	7.12627E-07	0.000378369	up	hypothetical protein DAI22_06g133650 [Oryza sativa Japonica Group]
Os11t0262600-00	4.278917211	0.000140543	0.023284055	up	hypothetical protein DAI22_11g096600 [Oryza sativa Japonica Group]
novel.468	4.098376448	4.19817E-07	0.000249318	up	hypothetical protein DAI22_04g035800 [Oryza sativa Japonica Group]

Os01t0728400-01	-7.017950892	5.7921E-07	0.000319407	down	Os01g0728400, partial [Oryza sativa Japonica Group]
Os06t0570566-00	-3.784462877	4.47231E-11	6.47395E-08	down	hypothetical protein EE612_034913 [Oryza sativa]
Os12t0637200-01	-2.988998116	3.30856E-11	5.10863E-08	down	hypothetical protein EE612_061148, partial [Oryza sativa]
Os06t0570600-00	-2.96179185	8.35082E-06	0.002511863	down	PUTATIVE PSEUDOGENE: RecName: Full=Ent-kaurene oxidase-like protein 1; Short=OsKOL1; AltName: Full=Cytochrome P450 701A7 [Oryza sativa Japonica Group]
Os01t0895200-01	-2.68484446	2.76958E-08	2.06924E-05	down	hypothetical protein EE612_007351 [Oryza sativa]
Os04t0481700-01	-2.673057256	0.000298866	0.040011764	down	MDR-like ABC transporter [Oryza sativa Japonica Group]
Os01t0930900-00	-2.599074007	0.000139142	0.023284055	down	tropinone reductase homolog At5g06060 [Oryza sativa Japonica Group]
Os03t0359600-01	-2.488459438	0.000322953	0.041968503	down	probable galacturonosyltransferase-like 4 [Oryza sativa Japonica Group]
Os02t0663900-01	-2.347373632	0.000233079	0.033118684	down	FT-interacting protein 1 [Oryza sativa Japonica Group]
Os01t0773400-00	-2.235602375	8.30333E-06	0.002511863	down	Os01g0773400 [Oryza sativa Japonica Group]
100MP$_S$_vs_0.5Cd-100MP$_S$					
ID	log2FoldChange	pvalue	padj	regulated	NR
Os12t0263800-03	7.585470999	5.81225E-09	1.24708E-06	up	hypothetical protein OsJ_17641 [Oryza sativa Japonica Group]
Os04t0125700-01	6.421567035	7.40187E-06	0.000687985	up	L-type lectin-domain containing receptor kinase IX.1 [Oryza sativa Japonica Group]
novel.151	6.172033423	3.10923E-05	0.002302637	up	hypothetical protein OsI_04686 [Oryza sativa Indica Group]
Os09t0241700-01	6.164779457	4.01135E-05	0.002851162	up	uncharacterized protein LOC112270781 [Brachypodium distachyon]
Os06t0110200-01	6.14983146	4.95335E-06	0.000498284	up	late embryogenesis abundant protein 6 [Oryza sativa Japonica Group]
Os08t0104400-01	5.910151827	9.27281E-21	1.52061E-17	up	uncharacterized protein LOC4344438 [Oryza sativa Japonica Group]
Os01t0738400-01	5.713835468	3.14326E-05	0.002312918	up	zinc finger CCCH domain-containing protein 10 [Oryza sativa Japonica Group]
novel.946	5.65220304	5.85839E-05	0.00385378	up	uncharacterized protein LOC107278025 [Oryza sativa Japonica Group]
Os12t0626500-00	5.569436325	2.06235E-20	2.78515E-17	up	late embryogenesis abundant protein D-34 [Oryza sativa Japonica Group]
Os04t0510900-01	5.53173854	4.696E-13	2.39579E-10	up	Os04g0510900, partial [Oryza sativa Japonica Group]
Os10t0403800-01	-6.461489137	0.000946655	0.031543243	down	transcription factor ILI3-like [Oryza sativa Japonica Group]
Os12t0104766-00	-6.224107319	0.0008502	0.029219908	down	hypothetical protein EE612_057292 [Oryza sativa]
Os10t0550800-00	-5.642160755	0.00054067	0.021587302	down	hypothetical protein EE612_052659, partial [Oryza sativa]
Os02t0777400-01	-5.503925563	0.000953703	0.031732048	down	ERECTA-like kinase [Oryza sativa Japonica Group]
Os05t0419800-01	-5.126770489	0.001801905	0.048367632	down	GDSL esterase/lipase At1g71691 [Oryza sativa Japonica Group]

Os07t0663800-01	-5.042987109	6.37147E-07	8.31114E-05	down	hypothetical protein EE612_041215, partial [Oryza sativa]
Os03t0343900-01	-4.415388306	1.92674E-06	0.000214728	down	hypothetical protein EE612_017344, partial [Oryza sativa]
Os11t0226800-00	-3.919389319	0.000741664	0.026856654	down	NBS-LRR-like protein [Oryza sativa Japonica Group]
Os10t0575600-01	-3.803699982	2.26128E-05	0.001790156	down	homeobox-leucine zipper protein ROC3 [Oryza sativa Japonica Group]
Os06t0316800-01	-3.725596561	0.000108186	0.006303879	down	glycine-rich RNA-binding protein 2 [Oryza sativa Japonica Group]
