# Supplementary material for: Phenotypic Changes and Physiological Genetic Responses of Oryza sativa L. Roots Under Stress of Nanoplastics (NPs) and Cadmium (Cd) in Single and Combination Forms
Source: Genes (Basel). 2026 Jul 21;17(7):835. doi: 10.3390/genes17070835 (PMC13409897; doi:10.3390/genes17070835)
Supplement: Supplementary file 1 [file genes-17-00835-s001.zip › Table S6.pdf]

Table S6 Eight co-regulated DEGs statistics

Gene	chr	start	end	description
novel.559	chr04	31807291	31808012	-
Os09t0499400-01	chr09	19329898	19331922	uncharacterized protein LOC4347475 [Oryza sativa Japonica Group]
Os07t0142700-01	chr07	2187378	2188973	uncharacterized acetyltransferase At3g50280 [Oryza sativa Japonica Group]
Os10t0454300-01	chr10	16599488	16600321	putative glycine-rich cell wall structural protein 1 [Oryza sativa Japonica Group]
Os10t0528400-01	chr10	20559589	20560614	uncharacterized protein LOC4349190 [Oryza sativa Japonica Group]
Os01t0155800-01	chr01	2999576	3001139	holotricin-3 [Oryza sativa Japonica Group]
Os10t0527800-01	chr10	20498004	20499096	uncharacterized protein LOC4349185 [Oryza sativa Japonica Group]
Os11t0226800-00	chr11	6631719	6636377	NBS-LRR-like protein [Oryza sativa Japonica Group]
