# Supplementary material for: Phenotypic Changes and Physiological Genetic Responses of Oryza sativa L. Roots Under Stress of Nanoplastics (NPs) and Cadmium (Cd) in Single and Combination Forms
Source: Genes (Basel). 2026 Jul 21;17(7):835. doi: 10.3390/genes17070835 (PMC13409897; doi:10.3390/genes17070835)
Supplement: Supplementary file 1 [file genes-17-00835-s001.zip › Table S7 .pdf]

Table S7 Verification of gene expression by qRT-PCR

Classification	ID	Primer orientation	primer
Glycine-rich RNA-binding proteins	Os12t0632000-01	Forward primer	GCTGAAGATGCGAAGGAGTT
		Reverse primer	CAGCTTCACGATGATGTTGG
Cell structure and cytoskeleton maintenance	Os11t0247300-01	Forward primer	TCATCGCCATCTTCTTCACC
		Reverse primer	GGTGATGAAGTAGTCGGTGC
	Os10t0454200-01	Forward primer	GACATGGAGAAGCTGGACAC
		Reverse primer	GTAGATCTTGAGGTCGGTGG
Protein folding and stress response	Os11t0703900-01	Forward primer	AAGCTGGTGAAGATGGACGA
		Reverse primer	TTGAGCTTGTCCAGGTAGGT
	Os09t0482100-01	Forward primer	ATGGAGCTGAGCAACAAGGT
		Reverse primer	ACGATGTTGTTCTCGATGCC
Redox and ROS scavenging	Os04t0689000-01	Forward primer	CGTGGATGAAGAGGTGGAGA
		Reverse primer	TGGTGATGGTGTTGTTGAGG
	Os03t0351500-01	Forward primer	AGAAGGACATCGACAAGCGT
		Reverse primer	TCTTCTTGTCCTCCATCGCT
	Os03t0285700-01	Forward primer	CTTCAAGTCGCTGCTGATGT
		Reverse primer	GAGTTCGATGTCCTTGGTGG
	Rice *Actin 1*	Forward primer	TGGCATCTCTCAGCACATTCC
		Reverse primer	TGCACAATGGATGGGTCAGA
