# Supplementary figures and images for: Phenotypic Changes and Physiological Genetic Responses of Oryza sativa L. Roots Under Stress of Nanoplastics (NPs) and Cadmium (Cd) in Single and Combination Forms
Source: Genes (Basel). 2026 Jul 21;17(7):835. doi: 10.3390/genes17070835 (PMC13409897; doi:10.3390/genes17070835)

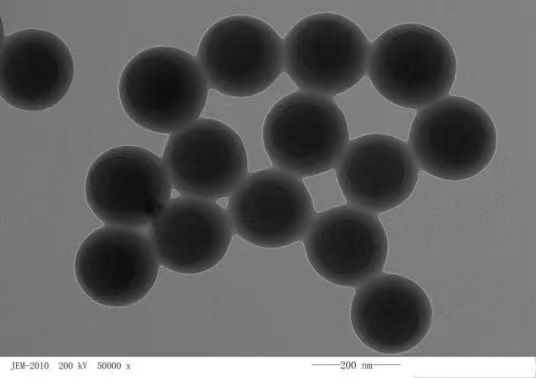

Supplement: Supplementary file 1 [file genes-17-00835-s001.zip › Figure S1.jpg]

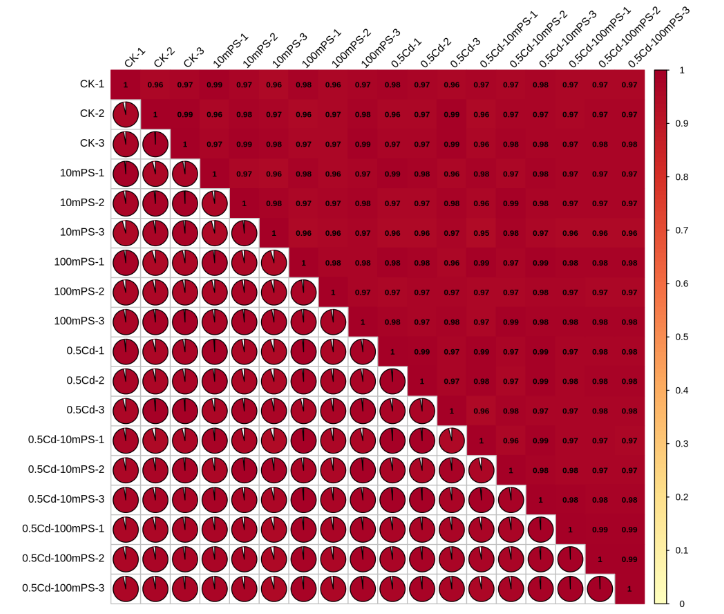

Supplement: Supplementary file 1 [file genes-17-00835-s001.zip › Figure S3.png]

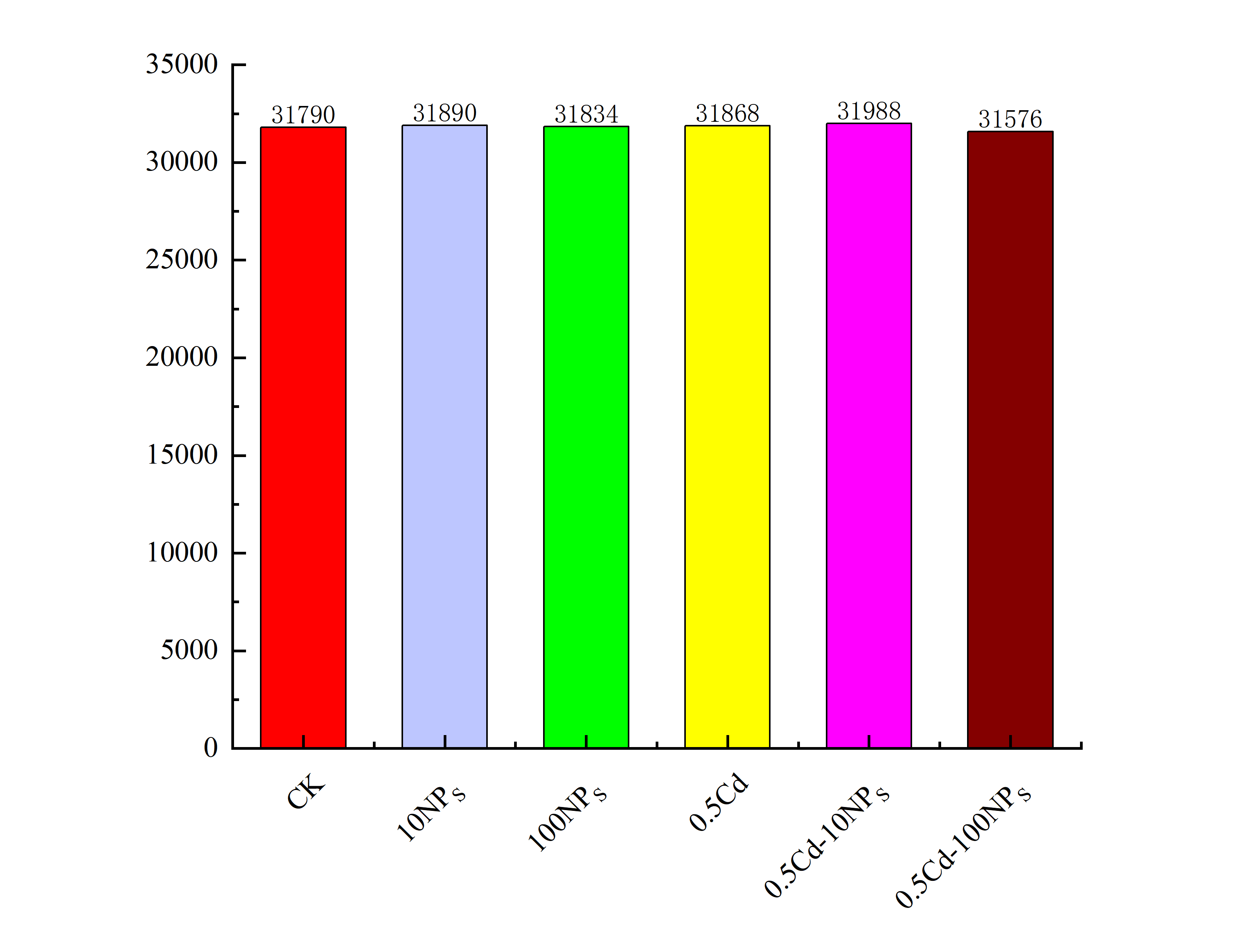

Supplement: Supplementary file 1 [file genes-17-00835-s001.zip › Figure S4.png]

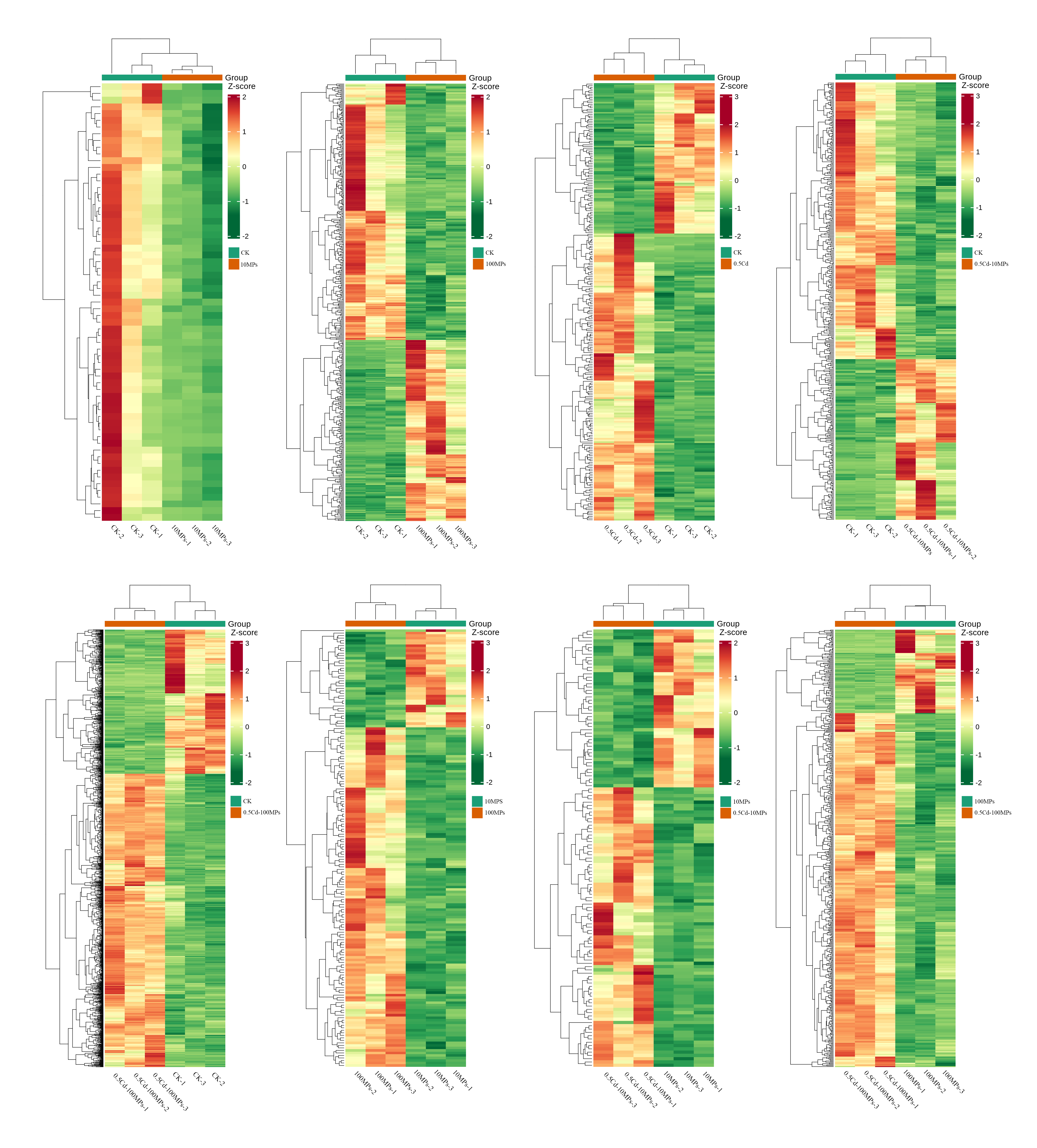

Supplement: Supplementary file 1 [file genes-17-00835-s001.zip › Figure S5.tif]
